# Regenerative endodontic procedures in immature permanent teeth with pulp necrosis: the impact of microbiology on clinical and radiographic outcome

**DOI:** 10.3389/fdmed.2023.1281337

**Published:** 2023-11-14

**Authors:** Rayann Sellami, Wannes Van Holm, Nastaran Meschi, Sarah Van Den Heuvel, Martine Pauwels, Tim Verspecht, Kathleen Vandamme, Wim Teughels, Paul Lambrechts

**Affiliations:** ^1^ Department of Oral Health Sciences, Endodontology, KU Leuven & Dentistry, University Hospitals Leuven, Leuven, Belgium; ^2^ Department of Oral Health Sciences, Periodontology, KU Leuven & Dentistry, University Hospitals Leuven, Leuven, Belgium; ^3^ Section of Endodontology, Department of Oral Health Sciences, Ghent University, Ghent, Belgium

**Keywords:** disinfection, quantitative polymerase chain reaction, microorganisms, root canal therapies, endodontics

## Abstract

**Aims:**

The current study aimed to determine how the disinfection strategy for regenerative endodontic procedures (REPs) influences overall bacterial load and REP outcomes. Different bacterial species in the teeth were also examined in this study.

**Methods:**

A previously reported non-randomized controlled clinical research on REP ± leukocyte and platelet-rich fibrin gathered microbial samples from 14 of 29 patients during REP (LPRF). Four microbiological samples were obtained in two treatment sessions. S1 and S2 were taken before and after the first irrigation with 1.5% NaOCl and saline. Samples S3 and S4 were obtained before and after rinsing with 17% EDTA in the second treatment session. Microbial samples were identified using a quantitative polymerase chain reaction with species-specific primers.

**Results:**

The total bacterial load recovered from patients showed a significant (*p* < 0.05) decrease after the first treatment and was maintained throughout the second treatment. *Fusobacterium nucleatum*, *Treponema denticola*, and *Enterococcus faecalis* were the most prevalent species in root canals, detected in all analyzed cases (100%), followed by *Prevotella intermedia* and *Tannerella forsythia*, both in six of 14 (42.9%) cases. The presence of these abundant species was significantly reduced after sample S1 was obtained. *Parvimonas micra* was present in four of 14 (28.6%) cases and *Actinomyces naeslundii* in two of 14 (14.3%) cases. *Filifactor alocis*, *Porphyromonas endodontalis*, and *Porphyromonas gingivalis* were each detected in only one of 14 (7.1%) cases. No statistical correlation could be made between bacterial species and clinical or radiographic outcomes due to the small sample size. In the LPRF group, two cases required retreatment due to early post-treatment flare-up, and two other cases presented radiographically presented a persistent apical periodontitis 3 years after treatment. In the control group, all analyzed cases were clinically asymptomatic after treatment, and radiographically, the final periapical index score at the last recall revealed healthy periapices.

**Conclusion:**

The REP disinfection protocol of the present study seems to be satisfactorily effective in reducing the total bacterial load, omitting clinical symptoms and inducing periapical bone healing in immature permanent teeth with pulp necrosis. However, LPRF seems to prevent these outcomes from being achieved and should consequently therefore not be recommended in REPs.

## 1. Introduction

Nowadays, even though tooth bioengineering is theoretically possible, we still do not know how to actually bioengineer a tooth to a specific size and shape (1, 2). However, when a tooth is still *in situ*, *de novo* regeneration of pulp and dentin can be attempted. With this possibility, the use of regenerative endodontic procedures (REPs) seems promising. REPs aim to restore the function of the damaged pulp-dentin complex by means of stem cells, scaffolds, and growth factors in an environment that is favorable for stem cell differentiation (3). Research has shown that inflammation, more specifically, a complement mechanism of fibroblasts of an injured pulp, aids regeneration (4). Nevertheless, it is also known that the presence of infectious agents negatively impacts stem cell differentiation, and the pulp fails to regenerate when infection persists (5). Furthermore, creating a sterile environment in the root canal system seems to be an endodontic impossibility, as even in a healthy, uninjured pulp-dentin complex, commensal micro-organisms are already present (6). Existing REP protocols (7, 8) provide recommendations for disinfection that is bactericidal on the one hand and non-lethal to the existing or transplanted stem cells on the other hand. Nevertheless, the recommendations regarding disinfection in REPs by the American Association of Endodontists (AAE) in 2016 were subsequently revised in 2018 and 2021. More specifically, it was stated that the previously recommended “light disinfection” with 1.5% sodium hypochlorite (NaOCl) (9) and a final concentration of 0.1–1 mg/ml double/triple antibiotic paste (10) would not sufficiently reduce the number of micro-organisms in order to prevent the residual bacteria from repopulating the unfilled root canal after REPs (7). After all, immature permanent teeth have thin root canal walls with larger dentinal tubules and a larger root canal in comparison with a mature permanent tooth. Hence, in the event of infection, there is physically more space for bacteria than in mature permanent teeth, and the microbial load can augment significantly (11).

In another study, the impact of the microbial load on the revitalization outcome was assessed (12). In that study, the clinical symptoms and the periapical lesions were successfully treated after REPs. Nevertheless, due to insufficient disinfection, there was a negative impact on the thickness of dentinal walls. Furthermore, as there is no mechanical debridement in REPs, an *in vitro* study reported the detrimental role of a residual biofilm on the release of TGF-β1 after dentin conditioning (13). In REP cases with a persistent infection, longer periods of disinfection lead to clinical success but histologically to repair rather than regenerate the pulp-dentin complex (14).

Treatment failures in REPs have been described previously (15, 16). On the one hand, failures in REPs occur due to the sequelae of trauma that cannot be impeded by current treatment protocols or other endodontic treatment modalities (15). On the other hand, failure due to a persistent infection also occurs (15), which indicates that the disinfection protocol of the current REPs is not always effective enough. The present study was performed to evaluate the impact of the REP disinfection protocol on the total bacterial load and how this affects the outcome of REPs in a previously reported controlled clinical trial (16).

## 2. Materials and methods

A brief REP protocol used by Meschi et al. (16) and the detailed microbial methods are mentioned below. This study was conducted according to the principles of Good Clinical Practice (International Council on Harmonization, 1996), which rely on the ethical principles of the Declaration of Helsinki (World Medical Association, 1964). This trial is registered under the following number: B322201421941.

### 2.1. REPs and microbial sample collection

In a previously reported non-randomized controlled clinical trial regarding REPs with (test group) and without (control group) leukocyte and platelet-rich fibrin (LPRF), microbial samples were collected during the REP. The operators were trained following a standardized procedure during the pilot phase of the study (16). The REP disinfection protocol in both groups was based on a study by Diogenes et al. (17). Briefly, local anesthesia with adrenalin was administered during the first treatment session. After rubber dam isolation, without operative field disinfection, and access cavity preparation, the working length (WL) determination procedure was performed using a periapical radiograph (PR) if no preoperative cone beam computed tomography (CBCT) was available. A microbial sample (S1) was taken with a sterile paper point at WL, and the paper point was stored in an Eppendorf with reduced transport fluid (RTF) at 4°C (18). Afterward, the root canal was irrigated 1 mm short of WL with 20 ml of 1.5% NaOCl and subsequently with 20 ml of saline. No inactivation of the irrigation solutions was performed. A second microbial sample (S2) was then taken at WL. The root canal was dried with sterile paper points, and calcium hydroxide (UltraCal™ XS; Ultradent Products, Inc., South Jordan, UT, USA) was injected into the root canal (1 mm short of WL). The tooth was temporarily sealed by means of a sterile cotton pellet and glass ionomer cement.

Two to four weeks later, the second session took place. Local anesthesia without adrenalin was administered, and the tooth was isolated by means of a rubber dam. After the removal of the temporary filling, a third microbial sample was taken (S3) by means of a sterile paper point at WL. After rinsing with 30 ml of EDTA 17% 1 mm short of WL, the fourth microbial sample was taken at WL (S4). In the test group, the exudate of an LPRF clot taken from the patient was used as a final rinse. Subsequently, in both groups, a blood clot was triggered periapically. In the control group, Collaplug (Zimmer Biomet, Berlin, Germany) was placed on the blood clot, and Pure Portland Cement Med-PZ (MPC; Medcem, Weinfelden, Switzerland) was used. The tooth was sealed by means of a glass ionomer lining and composite restoration. In the test group, LPRF clots and membranes were inserted into the root canal with endodontic pluggers up to 3 mm below the cementoenamel junction (CEJ). No Collaplug was applied below the MPC, as the LPRF scaffold provided enough resilience to the MPC.

For both groups, in case of flare-up, the treatment provided at the first session was repeated. Here again, pre- and post-disinfection microbial samples were taken at WL (S5 and S6). A double antibiotic paste (DAP; metronidazole 500 mg, ciprofloxacin 200 mg, Progel) was injected in the root canal instead of calcium hydroxide (17). However, the exact concentration of antibiotic paste was not measured. Furthermore, systemic antibiotics were also prescribed: amoxicillin or erythromycin (in case of penicillin allergy), dosed depending on the patient's weight.

For the patients treated at the university hospital (UZ Leuven, Leuven, Belgium), the Eppendorfs were immediately stored in a freezer at −20°C [Periodontology & Oral Microbiology (P&OM), KU Leuven, Leuven, Belgium]. The Eppendorfs containing the samples of the patients treated in private practice were stored in a portable freezer at −20°C (CoolFreeze CFX 35; Waeco, Emsdetten, Germany) and transported later for analysis.

The patients were followed up clinically and radiographically at 3, 6, 12, 24, and 36 months after the REP, the results of which are mentioned in Meschi et al. (16).

### 2.2. DNA extraction and quantitative polymerase chain reactions

DNA from microbiological samples was extracted using the QIAamp DNA Mini Kit® (QIAGEN®, Hilden, Germany) according to the manufacturer's instructions. Primers for *Actinomyces naeslundii*, *Fusobacterium nucleatum*, *Porphyromonas gingivalis*, and *Prevotella intermedia* were obtained from Slomka et al. (19). *Tannerella forsythia* primers were obtained from Boutaga et al. (20). *Filifactor alocis* was detected with PCRmax (Roche, Basel, Switzerland) primers and probes.

Primers for *Treponema denticola*, *Porphyromonas endodontalis*, *Enterococcus faecalis*, and *Parvimonas micra* were based on Nagata et al. (21) and redesigned by selecting sequences conserved in the available genomes for the species on GenBank® and blasted to not amplify other oral bacteria using BLASTN (22). Selected primers are presented in Table 1, and additional information on selection and primer properties is available in the Supplemental material (Appendix 1). Universal primers were not used due to their lack of sensitivity with a high limit of detection (LOD > 10,000 copies/ml) in no template controls.

**Table 1:** Species-specific qPCR primers used for bacterial cell quantification in microbiological samples.

Bacterial species	5′-3′ primers and probes (final concentration)	Amplicon size (bp)	Source
*Porphyromonas endodontalis*	Forward: GCTCAACTGTAGTCTTGCCGTTG (300 nm)	140	This study
	Reverse: GTGTCAGACGGAGCCTGGTAC (300 nm)		
*Parvimonas micra*	Forward: AGAGTTTGATCCTGGCTCAGGACG (300 nm)	117	This study
	Reverse: ACCCGTTCGCCACTTTCATTTCA (300 nm)		
*Treponema denticola*	Forward: GGTAAATGAGGAAAGGAGCTACGGC (300 nm)	100	This study
	Reverse: GGATACCCATCGTTGCCTTGGT (300 nm)		
*Enterococcus faecalis*	Forward: TCTTTCCTCCCGAGTGCTTGC (300 nm)	109	This study
	Reverse: AGCACCTGTTTCCAAGTGTTATCCC (300 nm)		
*Prevotella intermedia*	Forward: TGTGCCCYTTTGCATTTACCCTTC (300 nM)	216	Slomka et al. (19)
	Reverse: CACCATGAATTCCGCATACG (900 nM)		
	Probe: FAM-TGGCGGACTTGAGTGCACGC-TAMRA (200 nM)		
*Fusobacterium nucleatum*	Forward: GGATTTATTGGGCGTAAAGC (300 nM)	191	Slomka et al. (19)
	Reverse: ATCTGTCCAGTAAGCTGGCTTCC (300 nM)		
	Probe: FAM-CTCTACACTTGTAGTTCCG-TAMRA (300 nM)		
*Porphyromonas gingivalis*	Forward: CCGTAAGAATAAGCATCGGCTAACTC (300 nM)	195	Slomka et al. (19)
	Reverse: CACGAATTCCGCCTGC (300 nM)		
	Probe: FAM-CACTGAACTCAAGCCCGGCAGTTTCAA-TAMRA (100 nM)		
*Tannerella forsythia*	Forward: GGGTGAGTAACGCGTATGTAACCT (300 mM)	127	Honma et al. (23)
	Reverse: ACCCATCCGCAACCAATAAA (300 mM)		
	Probe: FAM-CCCGCAACAGAGGGATAACCCGG-TAMRA (100 nM)		
*Actinomyces naeslundii*	Forward: TCGAAACTCAGCAAGTAGCCG (200 nM)	155	Slomka et al. (19)
	Reverse: CGGAACAAACCTTTCCCAGGC (200 nM)		
	Probe: FAM-ATGAGTGGCGAACGGGTGAGTAAC-TAMRA (125 nM)		
*Filifactor alocis*	Forward: CAGGTGGTTTAACAAGTTAGTGG	594	Siqueira et al. (24)
	Reverse: CTAAGTTGTCCTTAGCTGTCTCG		
	Probe: FAM-TGG ATA CAG GTG GTG CAT GGT TGT-TAMRA		

Quantitative polymerase chain reactions (qPCRs) were performed with a CFX96 real-time system (Bio-Rad, Hercules, CA, USA), with reactions consisting of 12.5 µl of Takyon Rox probe master mix dTTP blue or Takyon™ Rox SYBR MasterMix dTTP Blue (Eurogentec, Seraing, Belgium), 1 µl of each primer (IDT, Haasrode, Belgium) and probe (all DD probes, 5′-FAM [6-carboxyfluorescein] and 3′-TAMRA [6-carboxytetramethylrhodamine]; Eurogentec, Seraing, Belgium), and 4.5 µl of Milli-Q water (or 5.5 µl for no probe). The cycling conditions were as follows: an initial step at 50°C for 2 min and 95°C for 10 min, followed by 45 cycles of 95°C for 15 s and 60°C for 1 min.

### 2.3. Statistical analysis

Statistical analysis was performed in R 4.0.0 (https://cran.r-project.org/). Data were analyzed with an ANOVA with Tukey HSD multiple comparisons (*p* < 0.05) to evaluate statistical differences in the bacterial species and the total bacterial load between sessions.

## 3. Results

**Figure 1:**
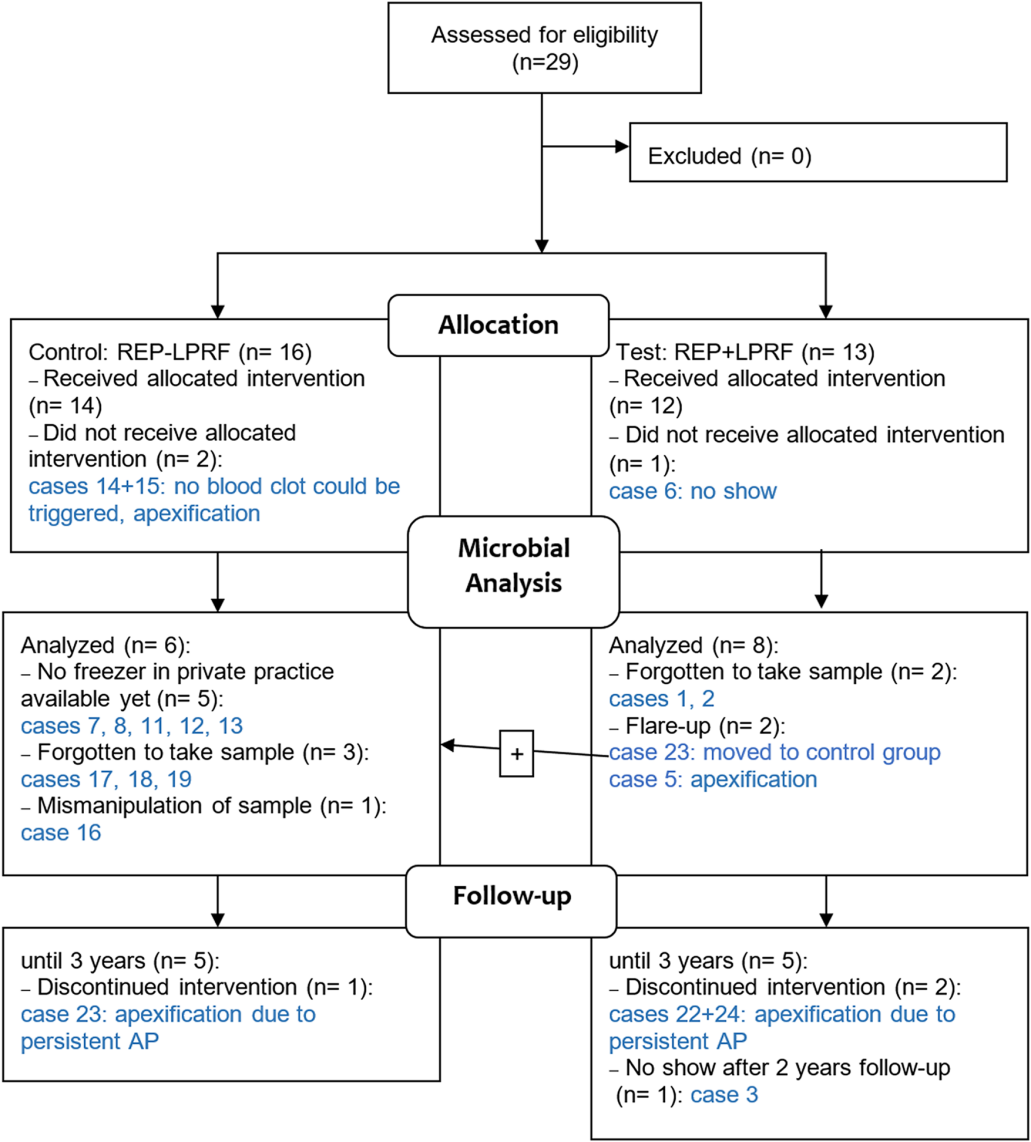
Flow diagram of the multicenter controlled clinical trial in REP ± LPRF with focus on the microbial analysis. The case numbers refer to the cases in Table 2. AP, apical periodontitis.

**Figure 2:**
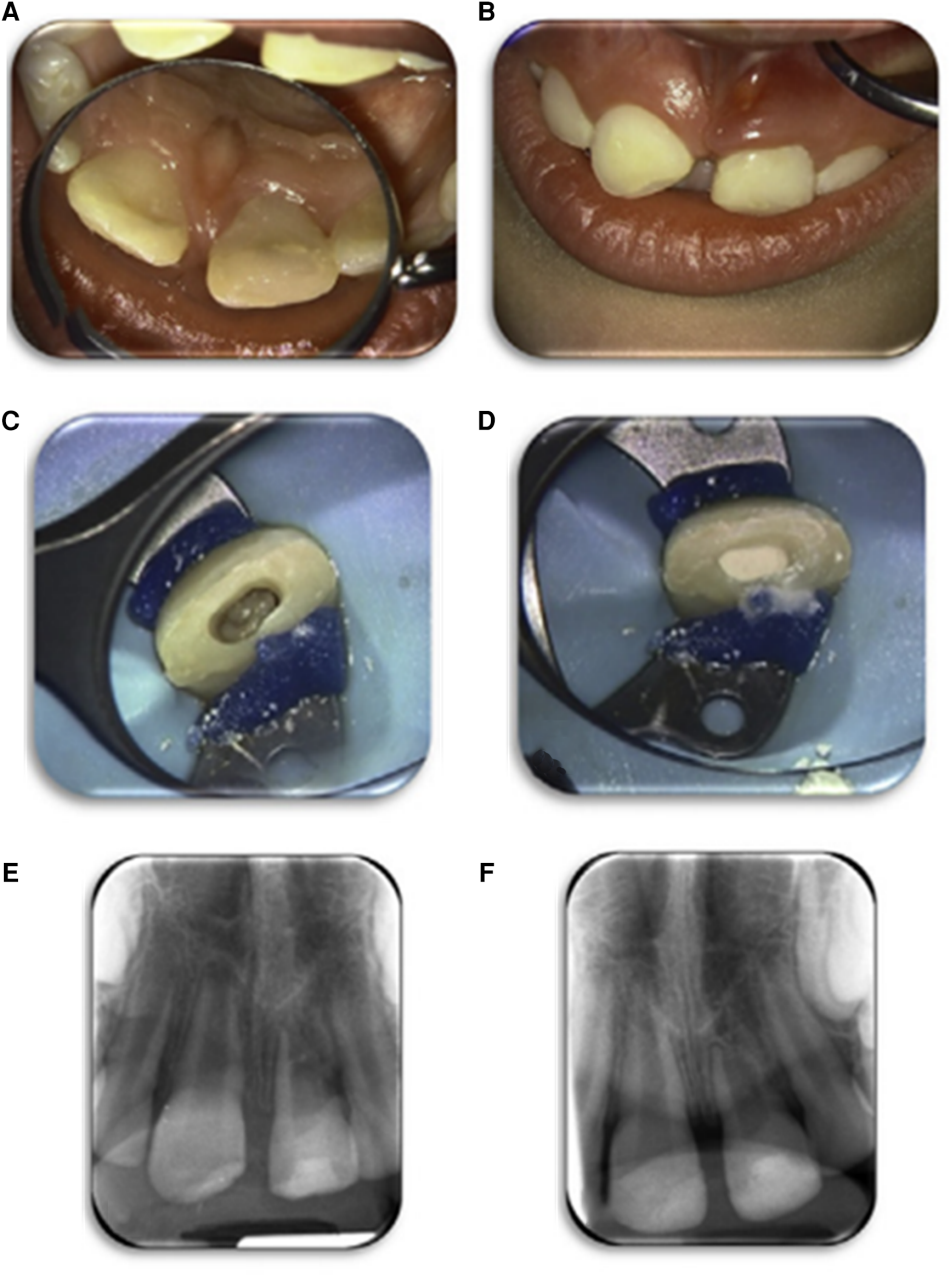
An example of a case of the REP (LPRF group). Case number 4 of Table 2. Clinical image before treatment of the left upper central incisor in the patient who received a REP treatment (**A**,**B**). The root canal was rinsed with an LPRF exudate; a periapical blood clot was triggered, and the root canal was filled with an LPRF clot (**C**). The LPRF was covered with Portland cement (Medcem), glass ionomer lining, and a composite restoration (**D**). Periapical radiographs: baseline (**E**) and follow-up 12 months (**F**). Images were repurposed by Meschi et al. (16), and their study contains a more thorough documentation of case 4.

The demographic data, clinical and radiographic outcomes, and total microbial load per case are provided in Table 2. From the 26 patients who received the allocated intervention (16) (Figure 1), only 14 were microbiologically analyzed, due to mainly lack of equipment and human errors, as shown in Figure 1, and representative dental photographs are presented in Figure 2.

**Table 2:** Demographic patient, clinical, radiographic, and microbial data of the controlled clinical trial [Meschi et al. (16)], obtained from only those patients in whom microbial samples were taken.

Case nr.		3	4	5	9	10	20	21	22	23	24	25	26	27	29
Sex, age (y)		M, 14	F, 7	F, 16	M, 8	F, 8	F, 11	M, 8	F, 8	F, 8	M, 12	F, 13	F, 10	M, 8	F, 8
Tooth		8	9	9	9	9	21	9	8	9	7	8	8	8	9
Etiology		Trauma	Trauma	DI	Trauma	Trauma	DI	Trauma	Trauma	Trauma	DI	Trauma	Trauma	Trauma	Trauma
Pre-op symptoms		Fistula	Fistula	Fistula	—	discoloration + abscess	Abscess	Fistula	Abscess	Abscess	Fistula	Abscess	Abscess	Abscess	Percussion pain
Pre-op periapical lesion		Yes	Yes	Yes	No	Yes	Yes	Yes	Yes	Yes	Yes	Yes	Yes	Yes	Yes
Last recall session (m)		24	37	n.a.	34	33	38	34	17	17	12	37	n.a.	36	36
Sensitivity post REP		2 y: EPT+, C-	All -	n.a.	3 y: EPT+	All-	All-	1 y: C+, EPT-	1 y: EPT+, C-	1 y: EPT+, C-	1 y: C+, EPT-	All-	n.a.	All-	1 y: EPT+, C+
Total microbial load	S1	3.89 × 10^6^	3.07 × 10^6^	7.72 × 10^8^	7.68 × 10^4^	1.85 × 10^5^	2.30 × 10^6^	9.59 × 10^5^	8.86 × 10^6^	2.32 × 10^6^	1.11 × 10^6^	5.01 × 10^7^	2.70 × 10^4^	1.19 × 10^5^	2.22 × 10^3^
	S2	3.05 × 10^4^	6.61 × 10^3^	5.61 × 10^4^	4.68 × 10^4^	1.74 × 10^3^	1.52 × 10^3^	2.37 × 10^3^	1.34 × 10^3^	2.70 × 10^4^	4.73 × 10^6^	2.60 × 10^2^	6.33 × 10^2^	3.01 × 10^2^	1.15 × 10^3^
	S3	1.34 × 10^4^	4.69 × 10^2^	3.07 × 10^3^	2.98 × 10^4^	3.10 × 10^3^	3.22 × 10^2^	0.00 × 10^0^	6.65 × 10^3^	6.53 × 10^3^	1.99 × 10^4^	2.71 × 10^2^	9.91 × 10^3^	3.91 × 10^2^	6.72 × 10^2^
	S4	4.07 × 10^3^	6.89 × 10^3^	2.83 × 10^4^	3.80 × 10^3^	2.06 × 10^3^	4.31 × 10^3^	1.01 × 10^4^	1.24 × 10^4^	3.37 × 10^4^	1.15 × 10^3^	1.94 × 10^2^	2.74 × 10^4^	3.14 × 10^2^	6.39 × 10^2^
3 D	Δ RHTV	11.6	−27.7	Flare-up 6 months post REP + apexification	No CBCT available	No CBCT available	13	No CBCT available	37.1	52.6	0.9	−7.8	No show 1-year post REP	38.8	31.2
	Δ RL	1.9	−27.8				1		−9.4	1.2	0	4.8		2	4.6
	Δ AA	0	490.9				−66.7		−100	−100	−47.1	−6.3		−100	−75.4
	Complete PBH (y)	Yes (2)	Yes (3)				Yes (3)		No (1)	No (1)	No (1)	No (3)		No (3)	Yes (3)
2 D	Δ RRA	−4	−6.7		5.5	−13	4.1	7.4	−10.5	95	41.9	19.8		4.7	40.4
	Δ RL	5.5	−23		10	−9.4	3.2	9.2	−1	−13.7	−8.8	4.5		8	32
	Final PAI (last recall)	1	1		1	1	2	1	2	1	4	2		1	1
	Healing type	1	^a^+^b^		1 + 4	2	2 + 5	3	^a^	^a^	1	^a^		2	1 + 2

The pulpal status of all cases was necrosis. Pink, test group; blue, control group.

AA, apical area; C, cold test; CBCT, cone beam computed tomography; D, dimensional; *Δ*, change (in %) between baseline and the last recall session; DI, dens invaginatus; EPT, electric pulp tester; F, female; M, male; m, month; NA, not applicable; PAI, periapical index; PBH, periapical bone healing; PR, periapical radiograph; REP, regenerative endodontic procedures; RHTV, root hard tissue volume; RL, root lengthening; RRA, radiographic root area; S1 microbial sample taken during first REP session prior to disinfection; S2 microbial sample taken during the first REP session after disinfection; S3 microbial sample taken during the second REP session prior to disinfection; S4 microbial sample taken during the first REP session after disinfection; y, year.

PAI final scores (Ørstavik D. et al., 1986), 1 and 2: success or healthy and 3–5: failure or diseased.

Types of REP healing as described by Chen et al., 2012) 1 = increased thickening of the root canal walls and continued root maturation, 2 = no significant continuation of root development with the root apex becoming blunt and closed, 3 = continued root development with the apical foramen remaining open, 4 = severe calcification (obliteration) of the root canal space; and 5 = a hard tissue barrier formed in the canal between the coronal biomaterial plug and the root apex.

^a^ No root development and no apical closure.

^b^ Ingrowth of hard tissue.

### 3.1. Microbial analysis

**Figure 3:**
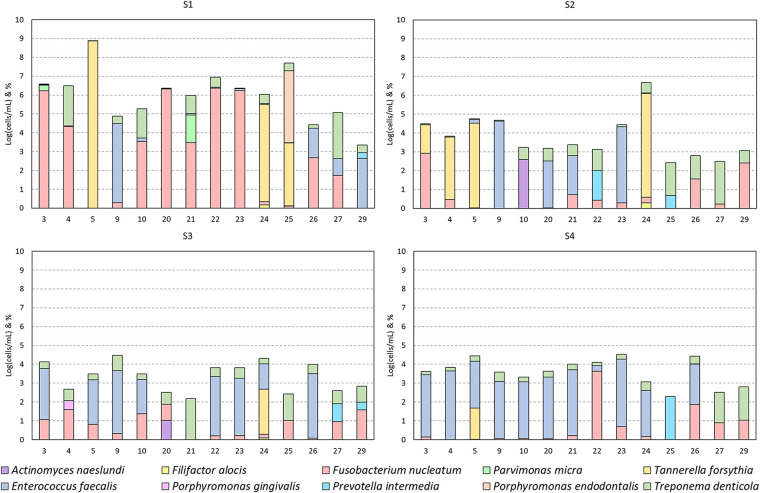
Bacterial detections grouped per session. Detections per case (numbers as mentioned in Table 2) presented as the base 10 logarithm of total cells per milliliter [log(cells/ml)] and the per species percentage composition of this total (ordered bottom-up). Data presented per patient in Figure 5.

In the different cases, diversity (=frequency) and abundance of the species mentioned in Figure 3 was noticeable (Figures 3 and 5). *F. nucleatum*, *T. denticola*, and *E. faecalis* were the most prevalent species in root canals, detected in all (100%) analyzed cases, followed by *P. intermedia* and *T. forsynthia*, both in six of 14 (42.85%) analyzed cases. *P. micra* was detected in four of 14 (28.57%) of the analyzed cases and *A. naeslundii* in two of the 14 (14.28%) cases. *F. alocis*, *P. endodontalis*, and *P. gingivalis* were each detected in only one of 14 (7.14%) cases.

Due to the low frequency of *A. naeslundii*, *F. alocis*, *P. gingivalis*, *P. intermedia*, and *P. endodontalis* and hence the low sample size, a statistical analysis of the effect of treatment was not performed for these species. However, if present, these species were usually only abundant in S1 and were either no longer present in subsequent sessions or at very low abundance (Figure 3).

The most abundant and frequent species in S1 was *F. nucleatum* [S1: 5.1 ± 1.7 log(cells/ml)]. This species was significantly reduced after the first treatment and remained low [average of S2, S3, and S4 combined: 2.27 ± 1.24 log(cells/ml); *p*-values: 0.0005, 0.021, and 0.0001, respectively]. Similarly, the second most abundant and frequent species, *T. denticola* [S1: 4.46 ± 1.61 log(cells/ml)], was also significantly reduced and remained low throughout the subsequent sessions [average of S2, S3, and S4 combined: 2.31 ± 1.13 log(cells/ml); *p*-values: 0.0003, 0.0011, and 0.0015, respectively].

*E. faecalis*, while not the most abundant species in S1, remained consistently present in some subjects but was significantly reduced in S2 and S3 [from S1: 4.13 ± 0.45 log(cells/ml) to 2.22 ± 1.89 log(cells/ml) in S2 and S3; *p*-values: 0.0008 and 0.0038, respectively], but not in S4 [not significant: remaining 2.88 ± 1.56 log(cells/ml)]. In S4, it was the most abundant and frequently present bacterium.

*T. forsythia*, while rare, was extremely abundant in case numbers 5, 24, and 25 before treatment [Table 2 and S1: 7.4 ± 1.19 log(cells/ml)]. After both sessions, it was only detected in case number 5 [from 8.8 log(cells/ml) in S1 to 4.03 log(cells/ml) in S4] and was not detectable in case numbers 24 and 25 (Figure 5).

*P. micra* was detected in four cases in S1 [4.43 ± 0.97 log(cells/ml)], but it was no longer present in S2–4.

**Figure 4:**
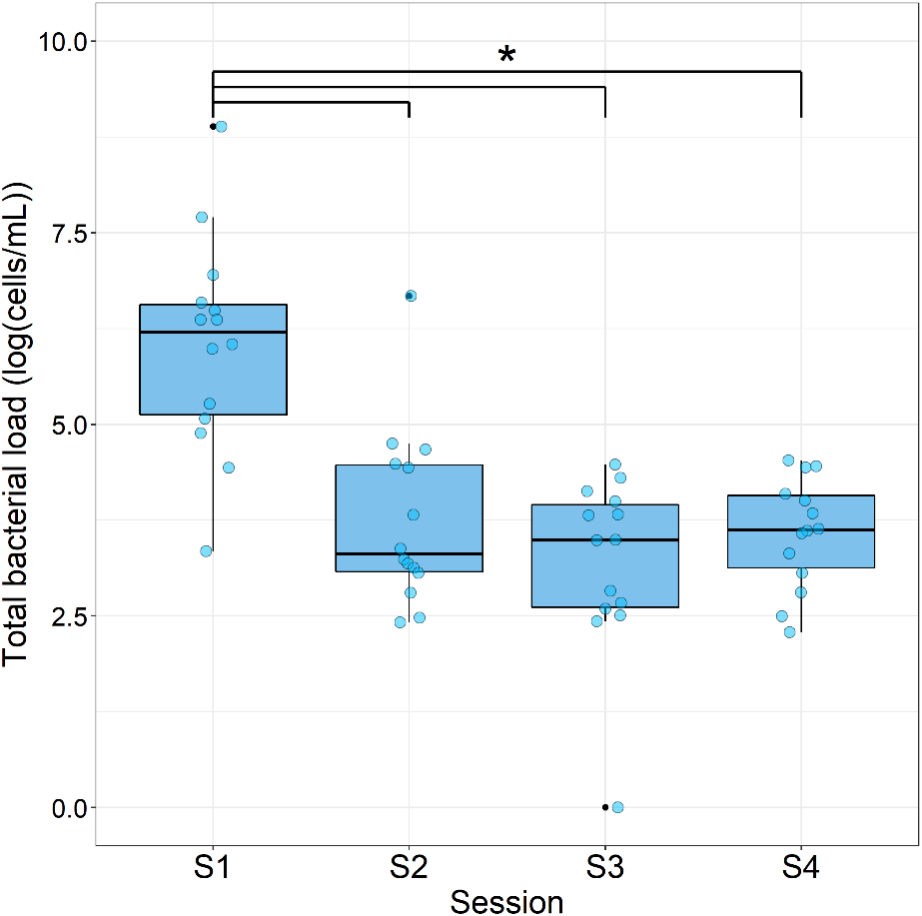
Total bacterial load during each sampling session. Data are expressed as box plots of the base 10 logarithm of the sum of bacterial cells per patient [log(cells/ml); *n* = 14]. * = significant differences (ANOVA with Tukey HSD; *p* < 0.05).

The total bacterial load, calculated as the sum of all bacterial detections per case, showed a significant (*p* < 0.05) decrease after the first treatment [Table 3, Figure 4; from S1: 6.03 ± 1.39 log(cells/ml) to 3.75 ± 1.15 log(cells/ml); *p*-value: <0.001]. This reduction in total bacterial load was maintained in S3 and S4 [3.18 ± 1.16 log(cells/ml); *p*-value: <0.001and 3.58 ± 0.72 log(cells/ml); *p*-value: 0.001, respectively].

**Table 3:** Confidence intervals and adjusted *p*-values from the ANOVA with Tukey HSD of the total biofilm loads between sessions.

	Lower	Upper	Adjusted *p*-value
S1–S2	1.142	3.410	<0.001
S1–S3	−3.979	−1.711	<0.001
S1–S4	1.311	3.579	<0.001
S2–S3	−1.703	0.565	0.547
S2–S4	−1.303	0.965	0.979
S3–S4	−1.534	0.734	0.786

### 3.2. Microbial load vs. clinical and radiographic outcome and survival

**Figure 5:**
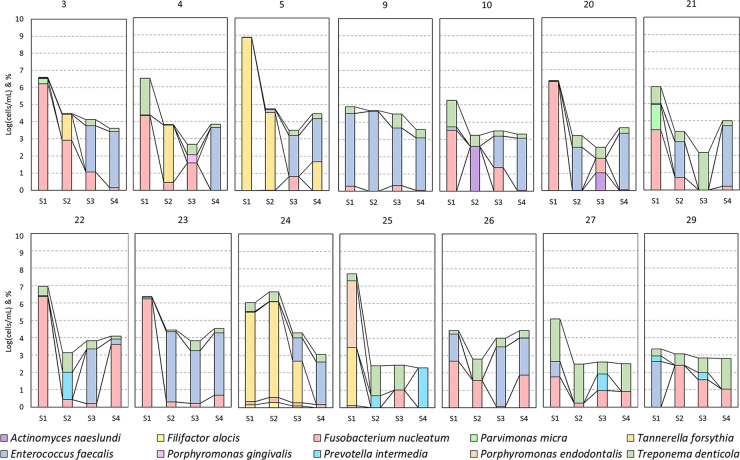
A per case (numbers as mentioned in Table 2) presentation of the bacterial detections of Figure 3.

Two cases (5 and 23) had a flare-up, and three cases (22, 23, and 24) had a persistent AP after REP + LPRF (Figure 1). Cases 22–24 had no substantial root lengthening (2D nor 3D) and qualitatively no further root development nor apical closure and survived due to apexification. Nevertheless, for all these four cases, a significant reduction in microbial load from S1 to S4 was detected (Table 2). For cases 5 and 24, *T. forsythia* was the most abundant species during the first session. For cases 5, 23, and 24, *E. faecalis* was prominent during the second treatment session (Figure 5).

For the test group cases (3, 4, 21, 25, and 27) with follow-up and without any complications, there was a substantial reduction of the microbial load from S1 to S4. Nevertheless, two of the five (40%) cases did not completely heal periapically after 3 years (on CBCT). The quantitative (2D and 3D) and qualitative (2D) assessments of further root development were inconsistent. For instance, two of the five (40%) cases (4 and 25) had a reduction in root hard tissue volume and in another two of the five (40%) cases (3 and 27) an increase (Table 2).

In addition, for the cases of the control group (9, 10, 20, and 29) with follow-up and without any complications, there was a substantial reduction of the microbial load from S1 to S4. Here all the cases had a final periapical index of 1, even if for case 9, *E. faecalis* remained abundant from S1 to S4. Except for case 10, there was an increase in root length and radiographic root area (2D), which was in accordance with the 3D measurements of cases with a CBCT after the REP (20 and 29).

In case 26, even if there was a reduction in the microbial load in S2 and S3, the total microbial load in S4 was more than in S1. Unfortunately, this patient was not compliant with recall appointments.

No correlation could be made between the etiology of pulp necrosis (trauma or dens invaginatus) and the microbial load.

## 4. Discussion

During a REP, the disinfectant should be bactericidal but simultaneously not lethal to mesenchymal stem cells (MSCs) (9, 25, 26), as they are indispensable for the further development of the immature permanent tooth. Keeping in mind that the root canal of an immature permanent tooth is a huge reservoir for potential bacteria and that some patients present late to the dentist, it is plausible that this fragile balance between disinfection and keeping MSCs alive is mostly at risk. Hence, the current study investigated the effectiveness of disinfection during REPs.

### 4.1. Generalizability

The most significant reduction in the total bacterial load was measured between S1 and S2; nevertheless, a continuing though smaller reduction occurred between the subsequent samples (Figure 4). This is in line with other similar studies (21, 27) and can be fully attributed to the antimicrobial properties of NaOCl during the first REP session (28). However, in the present study a concentration of only 1.5% NaOCl was used, and in the REP group in the study by Fouad et al. (27) only 1.25% NaOCl was used, in comparison with 6% in Nagata et al. (21). High concentrations of NaOCl might not be necessary to significantly reduce the total bacterial load in REP cases, which also favors MSC survival, as mentioned above (9, 23, 24). Nevertheless, in the study by Fouad et al. (27), 5.25% NaOCl was applied in the revascularization group, which presented significantly less final residual bacterial DNA than the REP group, which might impact early postoperative flare-ups (as was the case in the present study) and long-term further root development (13). Further root development did not occur in every case (Table 2), implying that root canal disinfection and the remaining bacterial load still play an important role in root development as also described in de-Jesus-Soares et al. (12).

Regarding the use of intracanalar antibiotic paste, it is noticeable that the concentration of triple antibiotic paste (TAP) used in Fouad et al. (27) in the revascularization group was tenfold that of the REP group in the same study. Furthermore, the REP group in that study had a substantial number of early clinical failures of treatment, which might have led to the recruitment discontinuation and thus a reduced sample size. This did not occur in the present study in the control group, but rather in the test group. More specifically, the addition of LPRF increased the risk of a postoperative flare-up and discontinued allocation concealment due to fear for venipuncture (Table 2) (16).

It has been reported that a decrease in bacterial load could occur by disrupting the bacterial membrane integrity through EDTA chelating properties (29). In the present study, 30 ml of 17% EDTA was used in the second REP session. Even if no significant decrease in bacterial load was measured between S3 and S4, the wash-out effect and the above-mentioned detrimental effect on the bacterial membrane might have caused the slight reduction in bacterial load (Figure 4).

Remarkably, the results of the present and previous studies favor single-visit REP, as the most significant reduction in total microbial load is seen after the first session. Treating and sealing the tooth in a single session also reduced the risk of intra- and inter-appointment microbial leakage due to fewer manipulations. However, a single-visit REP might not only be interesting from a microbiological point of view, but it also reduces the costs for and the need of compliance from, often young, patients. Even if the two-session REPs present higher success rates than the single appointment (30), this approach might have to be revisited in cases where no spontaneous inflammatory bleeding or pus exudate occurs during the first session.

The most prevalent species in all analyzed teeth were *F. nucleatum*, *T. denticola*, and *E. faecalis*, followed by *P. intermedia* and *T. forsynthia*. The prevalence of these anaerobic and facultative anaerobic species is in line with that of the literature (21, 27). For all the species investigated in the present study, there was a significant reduction after S1 (Figure 3). Nevertheless, the only abundant species in S4 was *E. faecalis* (Figure 3)*.* This species is known as one of the most aggressive and persistent species in endodontics, as it is not easily eradicated by the gold standard disinfectant, NaOCl (31). This could indicate that the REP disinfection protocol used is not effective enough against *E. faecalis*.

Regarding the use of scaffolds in REPs, it has been reported that morbidity caused by an infection could be reduced by the application of scaffolds acting as reservoirs for the long-term delivery of antimicrobials (such as silver nanoparticles, bacteriophages, or antimicrobial peptides) (32, 33). LPRF is described in the literature as a bioactive scaffold enhancing wound healing in terms of tissue regeneration. It releases its growth factors gradually over 7–14 days (34, 35). Nevertheless, in Meschi et al., for 25% of the patients in the REP + LPRF group, it has acted as an immunity bomb and resulted in a flare-up reaction in the early postoperative stage (16) (Figure 1). For the four cases (5, 22, 23, and 24, all in the REP + LPRF group) with a persistent AP, *E. faecalis* was the most abundant species in the second treatment session. The more prominent persistence of *E. faecalis* in cases 5 and 23 might have led to the failure of case 5 and the non-healing of the AP in case 23, even if additional disinfection during a re-REP took place. This was also applicable for case 26, as the microbial load at the end of the second session was even higher than at the start of treatment. Nevertheless, the significant reduction in the overall microbial load from S1 to S4 implies that not only residual bacteria in an “empty” root canal after REP but also the addition of LPRF has led to non-healing of the periapical lesions and flare-ups. It is noticeable noteworthy that such adverse events, occurring in a significant number of cases, have not been reported in other studies where autologous platelet concentrates have been applied in REPs (36–39). Furthermore, the non-healing of cases 22, 23, and 24 questions the positive reactions reported in sensitivity tests at the 1-year recall (Table 2).

### 4.2. Limitations

Logistical problems and human errors, as mentioned in Figure 1, reduced the sample size for the microbial analysis considerably. Hence, no correlation could be made between etiological factor (trauma or dens invaginatus) and microbial load. Nevertheless, despite this small sample size, the confidence intervals of S1–S2 and S1–S4 should suggest that even our small sample size had a significant change in load (Table 3).

A well-established protocol for microbial sampling and good manufacturing practices are not enough. As mentioned by Fouad et al., it is vital for training investigators to follow a standardized protocol to achieve an optimal clinical trial (27). While qPCR is a valuable tool for the accurate and inexpensive detection of specific bacteria, next-generation sequencing (NGS) approaches are becoming more accessible, allowing for the detection of every single microorganism without the need of specifically designed assays. NGS should be considered in future studies when researching the endodontic microbiome.

In a previous study, disinfecting the rubber dam and tooth (21, 27) and 1 ml 5% sodium thiosulfate were used to inactivate hypochlorite present before sampling (27). This was not done in the current trial, and no samples were taken of the teeth before access cavity preparation, which might have led to bias due to potential false-positive samples. It is essential to take a sterility sample before access preparation to rule out cases that should have been eliminated because of a positive sterility sample, as mentioned in Fouad et al. (27). Not inactivating the hypochlorite with sodium thiosulfate can result in sampling errors. Furthermore, operative field disinfection and inactivation of the irrigation solutions before sampling, as mentioned in previous studies (12, 21, 27), cannot be neglected in future clinical research. The main reason, why this was not done is that this was not mentioned in the clinical REP protocol of Diogenes et al. (17), which was the one applied in the current study. Later, in the ESE position statement (8), disinfecting the operating field was included but still not in the most recent version of the AAE clinical considerations on regenerative endodontic procedures (7).

On the one hand, an underdetection of bacteria might have occurred due to the sampling being performed by means of sterile paper points, even though this is the gold standard of sampling (40). Biofilms can only be partially disrupted by paper points in frequently difficult to access areas, which might limit the amount and consistency of the bacteria recovered. Sampling by means of K-files touching the root canal walls (without filing) could be an alternative, as reported in Fouad et al. Paper point samples could sample more planktonic bacteria than biofilms, thus resulting in sampling bias. A combination of paper points and K-files could be beneficial for future research to collect the residual bacteria on the K-files with other paper points (27). On the other hand, an overestimation of the residual bacteria might have taken place in the present study as well, as the DNA of killed bacteria was still counted as bacteria present even though their active pathogenesis and future proliferation should no longer be an issue (41). As in other studies that do not discount dead bacteria (12, 42), the present and future studies could benefit from other techniques such as viability qPCR, which removes DNA from dead bacteria from the equation (43).

Finally, only bacteria were researched in this study and not the full endodontic microbiome. In future endodontic microbiome studies, we should also focus on fungi and viruses. It is evident that they could also play a major role in the pathogenesis of apical periodontitis (AP). Herpes viruses and *Candida* species have been detected in cases of apical periodontitis, and their contribution remains to be clarified (44).

### 4.3. Interpretation and future challenges

The results of the present study indicate that the addition of the LPRF to REPs of infected teeth led to treatment failure in a significant number of cases. Proinflammatory cytokines in leukocytes might increase the inflammatory process during the early postoperative stage instead of enhancing the healing process (35). Hence, in future studies, this type of autologous platelet concentrate applied as a scaffold might be decisive for the long-term outcome of REPs.

If a REP in immature permanent teeth fails, it can be quite challenging to replace these teeth, as the main aim of REPs is to treat and preserve infected immature permanent teeth so that healthy bone is preserved for potential future implants. Controlling infection is the first hurdle to overcome in order to prolong the survival of REP-treated teeth before pulp regeneration can even begin (45). Strictly following the most recent evidence-based recommendations in dental trauma (https://dentaltraumaguide.org), the use of rubber dam isolation during treatment, achieving efficiency of disinfection, and placement of a tight coronal seal are crucial for optimal treatment outcomes.

Furthermore, the present study emphasizes that apexification is a treatment option to preserve teeth in case of REP failure due to a persistent periapical lesion. Finally, despite the limitations of the present study, for the cases patients in whom no without LPRF, the efficacy of the REP disinfection protocol resulted in periapical bone healing and further root development for a follow-up of 17–36 months.

## Data Availability

The raw data supporting the conclusions of this article will be made available by the authors, without undue reservation.
