# Supplementary material for: Regenerative endodontic procedures in immature permanent teeth with pulp necrosis: the impact of microbiology on clinical and radiographic outcome
Source: Front Dent Med. 2023 Nov 14;4:1281337. doi: 10.3389/fdmed.2023.1281337 (PMC11797765; doi:10.3389/fdmed.2023.1281337)
Supplement: Supplementary file 1 [file Datasheet1.docx]

**APPENDIX 1**

1. Primers

The forward and reverse primers for *Porphyromonas endodontalis*, *Parvimonas Micra*, *Treponema denticola* and *Enterococcus faecalis* were designed by Sellami R. and Drs. Van Holm W. The OligoAnalyser™ Tool from Integrated DNA Technologies, Inc was used. A target sequence with an amplicon length was chosen around 100 bp, with a GC content around 50-60% and no more than 3 consecutive repeats. The melting temperature (Tm) was designed to be between 60 and 64 °C with a maximum of 2 °C difference between reverse and forward primers. The primers were checked for secondary structures such as hairpins, self-dimers and hetero-dimers.^64^ The primer specificity was tested by blasting the primers and targeted amplicon lengths in BLAST®. *(Table 1)* Lastly all the available sp. strains of the researched bacteria in Genbank® for the targeted sites were checked. The primers were manufactured by Integrated DNA Technologies, Inc. The primers are given per species in *Table 2*.

| Bacterial species | Primers | Tm (°C) | Hairpin  (Delta G) | Selfdimer  (Delta G) | | Heterodimer  (Delta G) | Strains Blasted |
| --- | --- | --- | --- | --- | --- | --- | --- |
| *Porphyromonas endodontalis* | Forward  Reverse | 60  60 | -0.3  -0.25 | -5.2  -3.65 | -8  -8 | | ATCC 35406  NCTC13058 |
| *Parvimonas Micra* | Forward  Reverse | 62  62 | -2.5  0.6 | -8.2  -3.6 | -8.2  -8.2 | | NCTC11808  KCOM1535  KCOM1037  FDAARGOs_569  MGYG-HGUT-01301 |
| *Treponema denticola* | Forward  Reverse | 61  61 | -0.5  0.0 | -6.3  -5.0 | -6.6  -6.6 | | ATCC35405  AL2  ASLM  H-22  H1-T  SP23  ATCC 35404 |
| *Enterococcus faecalis* | Forward  Reverse | 61  61 | 0.0  -0.5 | -3.6  -3.3 | -8  -8 | | V583  VE14089  H25  CLB21560  BFFF11  TY1  KB1 |

*Table 1. Primer properties (OligoAnalyser™) and blasted strains in Blast® . A delta G > -9 is favorable*

*.*

| Bacterial species | Pairs of primers (5’-3’) | Amplicon Size (bp) | | Cycles |
| --- | --- | --- | --- | --- |
| *Porphyromonas endodontalis* | Forward: GCTCAACTGTAGTCTTGCCGTTG  Reverse: GTGTCAGACGGAGCCTGGTAC | 140 | Initial denaturation at 95°C for 10 min, followed by 45 cycles of 95°C for 15 sec, 60°C for 1 min. | |
| *Parvimonas micra* | Forward: AGAGTTTGATCCTGGCTCAGGACG  Reverse: ACCCGTTCGCCACTTTCATTTCA | 117 | Initial denaturation at 95°C for 10 min, followed by 45 cycles of 95°C for 15 sec, 60°C for 1 min. | |
| *Treponema denticola* | Forward: GGTAAATGAGGAAAGGAGCTACGGC  Reverse: GGATACCCATCGTTGCCTTGGT | 100 | Initial denaturation at 95°C for 10 min, followed by 45 cycles of 95°C for 15 sec, 60°C for 1 min. | |
| *Enterococcus faecalis* | Forward: TCTTTCCTCCCGAGTGCTTGC  Reverse: AGCACCTGTTTCCAAGTGTTATCCC | 109 | Initial denaturation at 95°C for 10 min, followed by 45 cycles of 95°C for 15 sec, 60°C for 1 min. | |

*Table 2. Quantitative PCR primers and length and cycling parameters for qPCR.*

1. Supplementary material

Unprocessed data

The investigators determined the number of bacteria/mL in the samples by means of qPCR. Standard curves were necessary for each bacterium to compare the samples. The standard curves with the equation, y = ax+b, were calculated after qPCR analysis. Different dilutions in a logarithmic scale of knowns quantity of bacteria DNA were used for the standard curves. This was done for each bacterium. Background noise of fluorescent signal occurred at most qPCR- analysis. The threshold line is set automatically by the software to separate the samples with background signal of fluoresce and samples containing the bacterium. The cycle quantification value or Cq-value stands for the PCR cycle number which the sample’s reaction curve intersect with the threshold line. The Cq-value tells how many cycles were needed to detect a real signal in the samples. The Cq value was obtained in a qPCR analysis by measuring the fluoresce.

The volume of template DNA used for the qPCR was 5µL. Initial sample volumes were 1000 µL before DNA-extraction. After DNA-extraction, the DNA of the samples was kept in the end volume of the DNA-extraction. The end volume was the elution buffer of 200 µL. The ribosomal copy numbers are different for each bacterium and were dependent on the used primers and binding places. The ribosomal copy numbers are given in *Table 3*.

| Bacterial species | Ribosomal copy numbers |
| --- | --- |
| *E. faecalis* | 4 |
| *T. denticola* | 2 |
| *P. micra* | 3 |
| *E. endodontalis*  *A.actinomycetemcomitans*  *P. gingivalis*  *P. intermedia*  *T. forsythia*  *F. nucleatum* | 1  6  4  3  2  5 |

*Table 3. Ribosomal copy numbers in the primers used for qPCR*

The numbers of copies per reaction or gene equivalent per µL were calculated by transforming the formula to x = ${10}^{\frac{Cq-b}{a}}$

To become the number of microorganisms per template, the number of detections per reaction was divided with the ribosomal copy number (*Table 3)*.

The calculation used for the number of microorganisms per mL was:

$$\frac{Number of MO}{mL}=\frac{number of MO}{template} * \frac{Initial sample Volume}{Volume of elution buffer} * \frac{1000}{Initial sample Volule}$$

In the following paragraphs, the standard set of dilutions and their associated standard curve with regression coefficient are given for each bacterium, along with the Cq-value, detections in template, number of MO in template, number of MO/mL for each sample and final MO/mL . Negative control samples consisted of distilled water and were labelled as “NTC” or No Template Control. Patient samples were labelled: “the number of the patient – treatment session – before/after disinfection”. The patient’s number was between 1 and 29. The treatment session is indicated with “a”, “b”, “c” or “d”, respectively for the first, second, third and fourth treatment session. Number “1” in subscript are the samples taken before disinfection, number “2” in subscript are the samples taken after disinfection.

The average of the NTC samples were used for the limit of detection. Sample values between the last limit of quantification and the limit of detection were replaced in the final results as value 0. The limit of quantification was the highest Cq-value from the standard curve used in the regression coefficient of that curve.

*2.1 Enterococcus faecalis*

*Standard dilutions and standard curve of E. faecalis*

| Log(copies/reaction) | Copies/reaction | Cq |
| --- | --- | --- |
| \| 1,01E+01 \| \| --- \| \| 9,08E+00 \| \| 8,08E+00 \| \| 7,08E+00 \| \| 6,08E+00 \| \| 5,08E+00 \| \| 4,08E+00 \| \| 3,08E+00 \| \| 2,08E+00 \| \| 1,08E+00 \| | \| 1,20E+10 \| \| --- \| \| 1,20E+09 \| \| 1,20E+08 \| \| 1,20E+07 \| \| 1,20E+06 \| \| 1,20E+05 \| \| 1,20E+04 \| \| 1,20E+03 \| \| 1,20E+02 \| \| 1,20E+01 \| | \| 19,67 \| \| --- \| \| 23,91 \| \| 28,06 \| \| 30,07 \| \| 35,99 \| \| 36,45 \| \| 40,94 \| \|  \| \|  \| \|  \| |

*Cq-value, detections in template, number of E. faecalis in template, number of E. faecalis/mL and final number of E. faecalis/mL*

| Sample | Cq | Detection in template | Number of MO in template | Number of MO/mL | Final number of MO/mL |
| --- | --- | --- | --- | --- | --- |
| NTC-01 | 43,49 | 0,00E+00 | 0,00E+00 | 0,00E+00 | 0,00E+00 |
| NTC-02 | 43,68 | 0,00E+00 | 0,00E+00 | 0,00E+00 | 0,00E+00 |
| 1a | 34,62 | 8,98E+05 | 2,25E+05 | 4,49E+04 | 4,49E+04 |
| 2a | 40,42 | 1,89E+04 | 4,72E+03 | 9,44E+02 | 9,44E+02 |
| 3a_1_ | 37,16 | 1,65E+05 | 4,14E+04 | 8,27E+03 | 8,27E+03 |
| 3a_2_ | 42,12 | 6,09E+03 | 1,52E+03 | 3,04E+02 | 0,00E+00 |
| 3b_1_ | 37,06 | 1,77E+05 | 4,42E+04 | 8,84E+03 | 8,84E+03 |
| 3b_2_ | 38,36 | 7,44E+04 | 1,86E+04 | 3,72E+03 | 3,72E+03 |
| 4a_1_ | 38,81 | 5,52E+04 | 1,38E+04 | 2,76E+03 | 2,76E+03 |
| 4a_2_ | 41,7 | 8,05E+03 | 2,01E+03 | 4,03E+02 | 0,00E+00 |
| 4b_1_ | N/A | 0,00E+00 | 0,00E+00 | 0,00E+00 | 0,00E+00 |
| 4b_2_ | 37,51 | 1,31E+05 | 3,28E+04 | 6,55E+03 | 6,55E+03 |
| 5a_1_ | 36,03 | 3,51E+05 | 8,78E+04 | 1,76E+04 | 1,76E+04 |
| 5a_2_ | 39,15 | 4,40E+04 | 1,10E+04 | 2,20E+03 | 2,20E+03 |
| 5b_1_ | 39,23 | 4,17E+04 | 1,04E+04 | 2,09E+03 | 2,09E+03 |
| 5b_2_ | 36,19 | 3,16E+05 | 7,89E+04 | 1,58E+04 | 1,58E+04 |
| 9a_1_ | 34,04 | 1,32E+06 | 3,30E+05 | 6,61E+04 | 6,61E+04 |
| 9a_2_ | 38,03 | 9,27E+04 | 2,32E+04 | 4,64E+03 | 4,64E+03 |
| 9b_1_ | 35,68 | 4,43E+05 | 1,11E+05 | 2,22E+04 | 2,22E+04 |
| 9b_2_ | 38,58 | 6,43E+04 | 1,61E+04 | 3,21E+03 | 3,21E+03 |
| 10a_1_ | 37,46 | 1,36E+05 | 3,39E+04 | 6,78E+03 | 6,78E+03 |
| 10a_2_ | 42,19 | 5,81E+03 | 1,45E+03 | 2,90E+02 | 0,00E+00 |
| 10b_1_ | 39,62 | 3,22E+04 | 8,04E+03 | 1,61E+03 | 1,61E+03 |
| 10b_2_ | 39,39 | 3,75E+04 | 9,37E+03 | 1,87E+03 | 1,87E+03 |
| 16a_1_ | 39,3 | 3,98E+04 | 9,95E+03 | 1,99E+03 | 1,99E+03 |
| 16a_2_ | 43,21 | 2,95E+03 | 7,36E+02 | 1,47E+02 | 0,00E+00 |
| 19a | 37,7 | 1,16E+05 | 2,89E+04 | 5,78E+03 | 5,78E+03 |
| 20a_1_ | 36,62 | 2,37E+05 | 5,93E+04 | 1,19E+04 | 1,19E+04 |
| 20a_2_ | 40,07 | 2,38E+04 | 5,96E+03 | 1,19E+03 | 1,19E+03 |
| 20b_1_ | N/A | 0,00E+00 | 0,00E+00 | 0,00E+00 | 0,00E+00 |
| 20b_2_ | 38,3 | 7,75E+04 | 1,94E+04 | 3,87E+03 | 3,87E+03 |
| 21a_1_ | 36,67 | 2,29E+05 | 5,73E+04 | 1,15E+04 | 1,15E+04 |
| 21a_2_ | 39,78 | 2,89E+04 | 7,23E+03 | 1,45E+03 | 1,45E+03 |
| 21b_1_ | N/A | 0,00E+00 | 0,00E+00 | 0,00E+00 | 0,00E+00 |
| 21b_2_ | 37,06 | 1,77E+05 | 4,42E+04 | 8,84E+03 | 8,84E+03 |
| 21c_1_ | 37,68 | 1,17E+05 | 2,93E+04 | 5,85E+03 | 5,85E+03 |
| 21c_2_ | 39,15 | 4,40E+04 | 1,10E+04 | 2,20E+03 | 2,20E+03 |
| 22a_1_ | 34,32 | 1,10E+06 | 2,74E+05 | 5,48E+04 | 5,48E+04 |
| 22a_2_ | 42,34 | 5,26E+03 | 1,31E+03 | 2,63E+02 | 0,00E+00 |
| 22b_2_ | 37,78 | 1,10E+05 | 2,74E+04 | 5,48E+03 | 5,48E+03 |
| 22b_1_ | 40,5 | 1,79E+04 | 4,48E+03 | 8,95E+02 | 8,95E+02 |
| 23a_1_ | 34,97 | 7,11E+05 | 1,78E+05 | 3,56E+04 | 3,56E+04 |
| 23a_2_ | 38,98 | 4,93E+04 | 1,23E+04 | 2,46E+03 | 2,46E+03 |
| 23b_1_ | 37,86 | 1,04E+05 | 2,60E+04 | 5,19E+03 | 5,19E+03 |
| 23b_2_ | 35,4 | 5,34E+05 | 1,34E+05 | 2,67E+04 | 2,67E+04 |
| 23c_2_ | 38,45 | 7,01E+04 | 1,75E+04 | 3,50E+03 | 3,50E+03 |
| 23d_2_ | 37,59 | 1,24E+05 | 3,11E+04 | 6,21E+03 | 6,21E+03 |
| 24a_1_ | 37,1 | 1,72E+05 | 4,31E+04 | 8,61E+03 | 8,61E+03 |
| 24a_2_ | 36,05 | 3,47E+05 | 8,66E+04 | 1,73E+04 | 1,73E+04 |
| 24b_1_ | 37,59 | 1,24E+05 | 3,11E+04 | 6,21E+03 | 6,21E+03 |
| 24b_2_ | 40,46 | 1,84E+04 | 4,60E+03 | 9,19E+02 | 9,19E+02 |
| 25a_1_ | 34,41 | 1,03E+06 | 2,58E+05 | 5,16E+04 | 5,16E+04 |
| 25a_2_ | 42,36 | 5,19E+03 | 1,30E+03 | 2,59E+02 | 0,00E+00 |
| 25b_1_ | 44,3 | 1,43E+03 | 3,56E+02 | 7,13E+01 | 0,00E+00 |
| 25c_1_ | 41,71 | 8,00E+03 | 2,00E+03 | 4,00E+02 | 0,00E+00 |
| 26a_1_ | 36,94 | 1,92E+05 | 4,79E+04 | 9,58E+03 | 9,58E+03 |
| 26a_2_ | 41,5 | 9,20E+03 | 2,30E+03 | 4,60E+02 | 0,00E+00 |
| 26b_1_ | 37,12 | 1,70E+05 | 4,25E+04 | 8,50E+03 | 8,50E+03 |
| 26b_2_ | 36,46 | 2,64E+05 | 6,59E+04 | 1,32E+04 | 1,32E+04 |
| 27a_1_ | 35,76 | 4,20E+05 | 1,05E+05 | 2,10E+04 | 2,10E+04 |
| 27a_2_ | 42,89 | 3,65E+03 | 9,11E+02 | 1,82E+02 | 0,00E+00 |
| 27b_1_ | N/A | 0,00E+00 | 0,00E+00 | 0,00E+00 | 0,00E+00 |
| 27b_2_ | N/A | 0,00E+00 | 0,00E+00 | 0,00E+00 | 0,00E+00 |
| 27c_2_ | 43,5 | 2,43E+03 | 6,07E+02 | 1,21E+02 | 0,00E+00 |
| 29a_1_ | 39,49 | 3,51E+04 | 8,77E+03 | 1,75E+03 | 1,75E+03 |
| 29a_2_ | 41,14 | 1,17E+04 | 2,92E+03 | 5,84E+02 | 0,00E+00 |
| 29b_1_ | 41,11 | 1,19E+04 | 2,98E+03 | 5,96E+02 | 0,00E+00 |
| 29b_2_ | 42,71 | 4,11E+03 | 1,03E+03 | 2,05E+02 | 0,00E+00 |

*2.2 Parvimonas micra*

*Standard dilutions and standard curve of* *P. micra*

| Log(copies/reaction) | Copies/reaction | Cq |
| --- | --- | --- |
| \| 1,09E+01 \| \| --- \| \| 9,86E+00 \| \| 8,86E+00 \| \| 7,86E+00 \| \| 6,86E+00 \| \| 5,86E+00 \| \| 4,86E+00 \| \| 3,86E+00 \| \| 2,86E+00 \| \| 1,86E+00 \| | \| 7,28E+10 \| \| --- \| \| 7,28E+09 \| \| 7,28E+08 \| \| 7,28E+07 \| \| 7,28E+06 \| \| 7,28E+05 \| \| 7,28E+04 \| \| 7,28E+03 \| \| 7,28E+02 \| \| 7,28E+01 \| | \| 11,04 \| \| --- \| \| 14,01 \| \| 17,69 \| \| 21,14 \| \| 24,71 \| \| 28,09 \| \| 31,37 \| \| 33,49 \| \|  \| \|  \| |

*Cq-value, detections in template, number of P. micra in template, number of P. micra /mL and final number of P. micra/mL*

| Sample | Cq | Detection in template | Number of MO in template | Number of MO/mL | Final number of MO/mL |
| --- | --- | --- | --- | --- | --- |
| NTC-01 | 39,37 | 0,00E+00 | 0,00E+00 | 0,00E+00 | 0,00E+00 |
| NTC-02 | 44,83 | 0,00E+00 | 0,00E+00 | 0,00E+00 | 0,00E+00 |
| 1a | 39,3 | 2,28E+02 | 7,58E+01 | 1,52E+01 | 0,00E+00 |
| 2a | 36,7 | 1,38E+03 | 4,61E+02 | 9,22E+01 | 0,00E+00 |
| 3a_1_ | 25,79 | 2,68E+06 | 8,94E+05 | 1,79E+05 | 1,79E+05 |
| 3a_2_ | 35,49 | 3,20E+03 | 1,07E+03 | 2,13E+02 | 0,00E+00 |
| 3b_1_ | 38,44 | 4,13E+02 | 1,38E+02 | 2,76E+01 | 0,00E+00 |
| 3b_2_ | 36,46 | 1,63E+03 | 5,44E+02 | 1,09E+02 | 0,00E+00 |
| 4a_1_ | 37,84 | 6,27E+02 | 2,09E+02 | 4,18E+01 | 0,00E+00 |
| 4a_2_ | 38,41 | 4,22E+02 | 1,41E+02 | 2,81E+01 | 0,00E+00 |
| 4b_1_ | 41,68 | 4,36E+01 | 1,45E+01 | 2,91E+00 | 0,00E+00 |
| 4b_2_ | 37,3 | 9,12E+02 | 3,04E+02 | 6,08E+01 | 0,00E+00 |
| 5a_1_ | 33,28 | 1,48E+04 | 4,95E+03 | 9,89E+02 | 9,89E+02 |
| 5a_2_ | 37,85 | 6,22E+02 | 2,07E+02 | 4,15E+01 | 0,00E+00 |
| 5b_1_ | 43,18 | 1,54E+01 | 5,14E+00 | 1,03E+00 | 0,00E+00 |
| 5b_2_ | 38,3 | 4,55E+02 | 1,52E+02 | 3,04E+01 | 0,00E+00 |
| 9a_1_ | 36,29 | 1,84E+03 | 6,12E+02 | 1,22E+02 | 0,00E+00 |
| 9a_2_ | N/A | 0,00E+00 | 0,00E+00 | 0,00E+00 | 0,00E+00 |
| 9b_1_ | 40,54 | 9,62E+01 | 3,21E+01 | 6,42E+00 | 0,00E+00 |
| 9b_2_ | 38,51 | 3,94E+02 | 1,31E+02 | 2,62E+01 | 0,00E+00 |
| 10a_1_ | 40,18 | 1,24E+02 | 4,12E+01 | 8,24E+00 | 0,00E+00 |
| 10a_2_ | 37,81 | 6,40E+02 | 2,13E+02 | 4,27E+01 | 0,00E+00 |
| 10b_1_ | 37,19 | 9,84E+02 | 3,28E+02 | 6,56E+01 | 0,00E+00 |
| 10b_2_ | 36,59 | 1,49E+03 | 4,97E+02 | 9,95E+01 | 0,00E+00 |
| 16a_1_ | 37,17 | 9,98E+02 | 3,33E+02 | 6,65E+01 | 0,00E+00 |
| 16a_2_ | 37,31 | 9,05E+02 | 3,02E+02 | 6,04E+01 | 0,00E+00 |
| 19a | 37,62 | 7,30E+02 | 2,43E+02 | 4,87E+01 | 0,00E+00 |
| 20a_1_ | 36,64 | 1,44E+03 | 4,80E+02 | 9,61E+01 | 0,00E+00 |
| 20a_2_ | 36,97 | 1,15E+03 | 3,82E+02 | 7,64E+01 | 0,00E+00 |
| 20b_1_ | 36,43 | 1,67E+03 | 5,56E+02 | 1,11E+02 | 0,00E+00 |
| 20b_2_ | 37,13 | 1,03E+03 | 3,42E+02 | 6,84E+01 | 0,00E+00 |
| 21a_1_ | 25,39 | 3,54E+06 | 1,18E+06 | 2,36E+05 | 2,36E+05 |
| 21a_2_ | 37,03 | 1,10E+03 | 3,67E+02 | 7,33E+01 | 0,00E+00 |
| 21b_1_ | 40,66 | 8,86E+01 | 2,95E+01 | 5,90E+00 | 0,00E+00 |
| 21b_2_ | 37,15 | 1,01E+03 | 3,37E+02 | 6,74E+01 | 0,00E+00 |
| 21c_1_ | 37,23 | 9,57E+02 | 3,19E+02 | 6,38E+01 | 0,00E+00 |
| 21c_2_ | 35,92 | 2,38E+03 | 7,92E+02 | 1,58E+02 | 0,00E+00 |
| 22a_1_ | 37,12 | 1,03E+03 | 3,44E+02 | 6,89E+01 | 0,00E+00 |
| 22a_2_ | 39,1 | 2,61E+02 | 8,71E+01 | 1,74E+01 | 0,00E+00 |
| 22b_2_ | 36,06 | 2,16E+03 | 7,18E+02 | 1,44E+02 | 0,00E+00 |
| 22b_1_ | 36,21 | 1,94E+03 | 6,47E+02 | 1,29E+02 | 0,00E+00 |
| 23a_1_ | 35,95 | 2,33E+03 | 7,75E+02 | 1,55E+02 | 0,00E+00 |
| 23a_2_ | 36,26 | 1,88E+03 | 6,25E+02 | 1,25E+02 | 0,00E+00 |
| 23b_1_ | 37,81 | 6,40E+02 | 2,13E+02 | 4,27E+01 | 0,00E+00 |
| 23b_2_ | 36,25 | 1,89E+03 | 6,30E+02 | 1,26E+02 | 0,00E+00 |
| 23c_2_ | 37,3 | 9,12E+02 | 3,04E+02 | 6,08E+01 | 0,00E+00 |
| 23d_2_ | 36,85 | 1,25E+03 | 4,15E+02 | 8,31E+01 | 0,00E+00 |
| 24a_1_ | 36,44 | 1,66E+03 | 5,52E+02 | 1,10E+02 | 0,00E+00 |
| 24a_2_ | 35,47 | 3,25E+03 | 1,08E+03 | 2,16E+02 | 0,00E+00 |
| 24b_1_ | 36,42 | 1,68E+03 | 5,60E+02 | 1,12E+02 | 0,00E+00 |
| 24b_2_ | 35,76 | 2,65E+03 | 8,85E+02 | 1,77E+02 | 0,00E+00 |
| 25a_1_ | 29,67 | 1,82E+05 | 6,06E+04 | 1,21E+04 | 1,21E+04 |
| 25a_2_ | 35,92 | 2,38E+03 | 7,92E+02 | 1,58E+02 | 0,00E+00 |
| 25b_1_ | 38,11 | 5,20E+02 | 1,73E+02 | 3,46E+01 | 0,00E+00 |
| 25c_1_ | 36,33 | 1,79E+03 | 5,96E+02 | 1,19E+02 | 0,00E+00 |
| 26a_1_ | 38,08 | 5,31E+02 | 1,77E+02 | 3,54E+01 | 0,00E+00 |
| 26a_2_ | 35,84 | 2,51E+03 | 8,37E+02 | 1,67E+02 | 0,00E+00 |
| 26b_1_ | 37,17 | 9,98E+02 | 3,33E+02 | 6,65E+01 | 0,00E+00 |
| 26b_2_ | 38,43 | 4,16E+02 | 1,39E+02 | 2,77E+01 | 0,00E+00 |
| 27a_1_ | 37,04 | 1,09E+03 | 3,64E+02 | 7,28E+01 | 0,00E+00 |
| 27a_2_ | 39,02 | 2,76E+02 | 9,21E+01 | 1,84E+01 | 0,00E+00 |
| 27b_1_ | 35,69 | 2,79E+03 | 9,29E+02 | 1,86E+02 | 0,00E+00 |
| 27b_2_ | 36,44 | 1,66E+03 | 5,52E+02 | 1,10E+02 | 0,00E+00 |
| 27c_2_ | 35,75 | 2,67E+03 | 8,91E+02 | 1,78E+02 | 0,00E+00 |
| 29a_1_ | 36,4 | 1,70E+03 | 5,67E+02 | 1,13E+02 | 0,00E+00 |
| 29a_2_ | 38,42 | 4,19E+02 | 1,40E+02 | 2,79E+01 | 0,00E+00 |
| 29b_1_ | 35,96 | 2,31E+03 | 7,70E+02 | 1,54E+02 | 0,00E+00 |
| 29b_2_ | 37,52 | 7,83E+02 | 2,61E+02 | 5,22E+01 | 0,00E+00 |

*2.3 Porphyromonas endodontalis*

*Standard dilutions and standard curve of P. endodontalis*

| Log(copies/reaction) | Copies/reaction | Cq |
| --- | --- | --- |
| \| 1,09E+01 \| \| --- \| \| 9,92E+00 \| \| 8,92E+00 \| \| 7,92E+00 \| \| 6,92E+00 \| \| 5,92E+00 \| \| 4,92E+00 \| \| 3,92E+00 \| \| 2,92E+00 \| \| 1,92E+00 \| | \| 8,36E+10 \| \| --- \| \| 8,36E+09 \| \| 8,36E+08 \| \| 8,36E+07 \| \| 8,36E+06 \| \| 8,36E+05 \| \| 8,36E+04 \| \| 8,36E+03 \| \| 8,36E+02 \| \| 8,36E+01 \| | \| 10,62 \| \| --- \| \| 14,2 \| \| 17,99 \| \| 21,24 \| \| 23,55 \| \| 26,96 \| \| 31,45 \| \|  \| \|  \| \|  \| |

*Cq-value, detections in template, number of P. endodontalis in template, number of P. endodontalis /mL and final number of P. endodontalis/mL*

| Sample | Cq | Detection in template | Number of MO in template | Number of MO/mL | Final number of MO/mL |
| --- | --- | --- | --- | --- | --- |
| NTC-01 | 36,46 | 0,00E+00 | 0,00E+00 | 0,00E+00 | 0,00E+00 |
| NTC-02 | 38,59 | 0,00E+00 | 0,00E+00 | 0,00E+00 | 0,00E+00 |
| 1a | 35,21 | 4,24E+03 | 4,24E+03 | 8,49E+02 | 0,00E+00 |
| 2a | 37,02 | 1,22E+03 | 1,22E+03 | 2,44E+02 | 0,00E+00 |
| 3a_1_ | 35,91 | 2,62E+03 | 2,62E+03 | 5,24E+02 | 0,00E+00 |
| 3a_2_ | 34,64 | 6,28E+03 | 6,28E+03 | 1,26E+03 | 0,00E+00 |
| 3b_1_ | 37,19 | 1,08E+03 | 1,08E+03 | 2,17E+02 | 0,00E+00 |
| 3b_2_ | 40,56 | 1,06E+02 | 1,06E+02 | 2,13E+01 | 0,00E+00 |
| 4a_1_ | 36,45 | 1,81E+03 | 1,81E+03 | 3,61E+02 | 0,00E+00 |
| 4a_2_ | 37,31 | 9,98E+02 | 9,98E+02 | 2,00E+02 | 0,00E+00 |
| 4b_1_ | 36,04 | 2,40E+03 | 2,40E+03 | 4,79E+02 | 0,00E+00 |
| 4b_2_ | 37,5 | 8,76E+02 | 8,76E+02 | 1,75E+02 | 0,00E+00 |
| 5a_1_ | 35,35 | 3,85E+03 | 3,85E+03 | 7,71E+02 | 0,00E+00 |
| 5a_2_ | 38,77 | 3,65E+02 | 3,65E+02 | 7,30E+01 | 0,00E+00 |
| 5b_1_ | 36,9 | 1,32E+03 | 1,32E+03 | 2,65E+02 | 0,00E+00 |
| 5b_2_ | 37,07 | 1,18E+03 | 1,18E+03 | 2,36E+02 | 0,00E+00 |
| 9a_1_ | 33,77 | 1,14E+04 | 1,14E+04 | 2,29E+03 | 0,00E+00 |
| 9a_2_ | 40,36 | 1,22E+02 | 1,22E+02 | 2,44E+01 | 0,00E+00 |
| 9b_1_ | 36,07 | 2,35E+03 | 2,35E+03 | 4,69E+02 | 0,00E+00 |
| 9b_2_ | 39,24 | 2,64E+02 | 2,64E+02 | 5,28E+01 | 0,00E+00 |
| 10a_1_ | 36,83 | 1,39E+03 | 1,39E+03 | 2,78E+02 | 0,00E+00 |
| 10a_2_ | 36,51 | 1,73E+03 | 1,73E+03 | 3,47E+02 | 0,00E+00 |
| 10b_1_ | 40,48 | 1,12E+02 | 1,12E+02 | 2,25E+01 | 0,00E+00 |
| 10b_2_ | 37,59 | 8,23E+02 | 8,23E+02 | 1,65E+02 | 0,00E+00 |
| 16a_1_ | 37,71 | 7,58E+02 | 7,58E+02 | 1,52E+02 | 0,00E+00 |
| 16a_2_ | 37,93 | 6,51E+02 | 6,51E+02 | 1,30E+02 | 0,00E+00 |
| 19a | 36,63 | 1,60E+03 | 1,60E+03 | 3,19E+02 | 0,00E+00 |
| 20a_1_ | 37,7 | 7,63E+02 | 7,63E+02 | 1,53E+02 | 0,00E+00 |
| 20a_2_ | 39,31 | 2,52E+02 | 2,52E+02 | 5,03E+01 | 0,00E+00 |
| 20b_1_ | 42,13 | 3,61E+01 | 3,61E+01 | 7,21E+00 | 0,00E+00 |
| 20b_2_ | 39,24 | 2,64E+02 | 2,64E+02 | 5,28E+01 | 0,00E+00 |
| 21a_1_ | 38,36 | 4,84E+02 | 4,84E+02 | 9,69E+01 | 0,00E+00 |
| 21a_2_ | 36,75 | 1,47E+03 | 1,47E+03 | 2,94E+02 | 0,00E+00 |
| 21b_1_ | 41,11 | 7,28E+01 | 7,28E+01 | 1,46E+01 | 0,00E+00 |
| 21b_2_ | 36,44 | 1,82E+03 | 1,82E+03 | 3,64E+02 | 0,00E+00 |
| 21c_1_ | 38,29 | 5,08E+02 | 5,08E+02 | 1,02E+02 | 0,00E+00 |
| 21c_2_ | 39,05 | 3,01E+02 | 3,01E+02 | 6,02E+01 | 0,00E+00 |
| 22a_1_ | 35,28 | 4,04E+03 | 4,04E+03 | 8,09E+02 | 0,00E+00 |
| 22a_2_ | 36,65 | 1,57E+03 | 1,57E+03 | 3,15E+02 | 0,00E+00 |
| 22b_2_ | 37,48 | 8,88E+02 | 8,88E+02 | 1,78E+02 | 0,00E+00 |
| 22b_1_ | 40,06 | 1,50E+02 | 1,50E+02 | 3,00E+01 | 0,00E+00 |
| 23a_1_ | 36,68 | 1,54E+03 | 1,54E+03 | 3,08E+02 | 0,00E+00 |
| 23a_2_ | 37,14 | 1,12E+03 | 1,12E+03 | 2,24E+02 | 0,00E+00 |
| 23b_1_ | 38,03 | 6,08E+02 | 6,08E+02 | 1,22E+02 | 0,00E+00 |
| 23b_2_ | 36,32 | 1,97E+03 | 1,97E+03 | 3,95E+02 | 0,00E+00 |
| 23c_2_ | 38,34 | 4,91E+02 | 4,91E+02 | 9,82E+01 | 0,00E+00 |
| 23d_2_ | 38,03 | 6,08E+02 | 6,08E+02 | 1,22E+02 | 0,00E+00 |
| 24a_1_ | 35,44 | 3,62E+03 | 3,62E+03 | 7,24E+02 | 0,00E+00 |
| 24a_2_ | 36,29 | 2,02E+03 | 2,02E+03 | 4,03E+02 | 0,00E+00 |
| 24b_1_ | 36,74 | 1,48E+03 | 1,48E+03 | 2,96E+02 | 0,00E+00 |
| 24b_2_ | 37,01 | 1,23E+03 | 1,23E+03 | 2,46E+02 | 0,00E+00 |
| 25a_1_ | 20,28 | 1,25E+08 | 1,25E+08 | 2,49E+07 | 2,49E+07 |
| 25a_2_ | 38,52 | 4,34E+02 | 4,34E+02 | 8,67E+01 | 0,00E+00 |
| 25b_1_ | 36,23 | 2,10E+03 | 2,10E+03 | 4,20E+02 | 0,00E+00 |
| 25c_1_ | 37,1 | 1,15E+03 | 1,15E+03 | 2,31E+02 | 0,00E+00 |
| 26a_1_ | 36,71 | 1,51E+03 | 1,51E+03 | 3,02E+02 | 0,00E+00 |
| 26a_2_ | 39,16 | 2,79E+02 | 2,79E+02 | 5,58E+01 | 0,00E+00 |
| 26b_1_ | 37,22 | 1,06E+03 | 1,06E+03 | 2,12E+02 | 0,00E+00 |
| 26b_2_ | 36,32 | 1,97E+03 | 1,97E+03 | 3,95E+02 | 0,00E+00 |
| 27a_1_ | 35,99 | 2,48E+03 | 2,48E+03 | 4,96E+02 | 0,00E+00 |
| 27a_2_ | 36,41 | 1,86E+03 | 1,86E+03 | 3,71E+02 | 0,00E+00 |
| 27b_1_ | 38,2 | 5,41E+02 | 5,41E+02 | 1,08E+02 | 0,00E+00 |
| 27b_2_ | 41,9 | 4,22E+01 | 4,22E+01 | 8,45E+00 | 0,00E+00 |
| 27c_2_ | 38,68 | 3,88E+02 | 3,88E+02 | 7,77E+01 | 0,00E+00 |
| 29a_1_ | 40,05 | 1,51E+02 | 1,51E+02 | 3,02E+01 | 0,00E+00 |
| 29a_2_ | 41,69 | 4,88E+01 | 4,88E+01 | 9,76E+00 | 0,00E+00 |
| 29b_1_ | 40,53 | 1,09E+02 | 1,09E+02 | 2,17E+01 | 0,00E+00 |
| 29b_2_ | 39,06 | 2,99E+02 | 2,99E+02 | 5,98E+01 | 0,00E+00 |

*2.4 Treponema denticola*

*Standard dilutions and standard curve of T. denticola*

| Log(copies/reaction) | Copies/reaction | Cq |
| --- | --- | --- |
| \| 1,04E+01 \| \| --- \| \| 9,45E+00 \| \| 8,45E+00 \| \| 7,45E+00 \| \| 6,45E+00 \| \| 5,45E+00 \| \| 4,45E+00 \| \| 3,45E+00 \| \| 2,45E+00 \| \| 1,45E+00 \| | \| 2,79E+10 \| \| --- \| \| 2,79E+09 \| \| 2,79E+08 \| \| 2,79E+07 \| \| 2,79E+06 \| \| 2,79E+05 \| \| 2,79E+04 \| \| 2,79E+03 \| \| 2,79E+02 \| \| 2,79E+01 \| | \| 20,13 \| \| --- \| \| 22,82 \| \| 25,53 \| \| 29,1 \| \|  \| \| 35,95 \| \|  \| \|  \| \|  \| \|  \| |

*Cq-value, detections in template, number of T. denticola in template, number of T. denticola/mL and final number of T. denticola/mL.*

| Sample | Cq | Detection in template | Number of MO in template | Number of MO/mL | Final number of MO/mL |
| --- | --- | --- | --- | --- | --- |
| NTC-01 | 38,3 | 0,00E+00 | 0,00E+00 | 0,00E+00 | 0,00E+00 |
| NTC-02 | 38,48 | 0,00E+00 | 0,00E+00 | 0,00E+00 | 0,00E+00 |
| 1a | 27,59 | 9,29E+07 | 4,65E+07 | 9,29E+06 | 9,29E+06 |
| 2a | 31,58 | 5,20E+06 | 2,60E+06 | 5,20E+05 | 5,20E+05 |
| 3a_1_ | 29,53 | 2,29E+07 | 1,14E+07 | 2,29E+06 | 2,29E+06 |
| 3a_2_ | 35,26 | 3,64E+05 | 1,82E+05 | 3,64E+04 | 3,64E+04 |
| 3b_1_ | 33,44 | 1,36E+06 | 6,78E+05 | 1,36E+05 | 1,36E+05 |
| 3b_2_ | 35,86 | 2,36E+05 | 1,18E+05 | 2,36E+04 | 2,36E+04 |
| 4a_1_ | 24,25 | 1,04E+09 | 5,19E+08 | 1,04E+08 | 1,04E+08 |
| 4a_2_ | 37,15 | 9,30E+04 | 4,65E+04 | 9,30E+03 | 0,00E+00 |
| 4b_1_ | 36,67 | 1,32E+05 | 6,58E+04 | 1,32E+04 | 0,00E+00 |
| 4b_2_ | 35,12 | 4,03E+05 | 2,02E+05 | 4,03E+04 | 4,03E+04 |
| 5a_1_ | 23,6 | 1,66E+09 | 8,30E+08 | 1,66E+08 | 1,66E+08 |
| 5a_2_ | 34,47 | 6,45E+05 | 3,22E+05 | 6,45E+04 | 6,45E+04 |
| 5b_1_ | 35,43 | 3,22E+05 | 1,61E+05 | 3,22E+04 | 3,22E+04 |
| 5b_2_ | 32,82 | 2,12E+06 | 1,06E+06 | 2,12E+05 | 2,12E+05 |
| 9a_1_ | 31,16 | 7,05E+06 | 3,52E+06 | 7,05E+05 | 7,05E+05 |
| 9a_2_ | 34,77 | 5,19E+05 | 2,59E+05 | 5,19E+04 | 5,19E+04 |
| 9b_1_ | 31,33 | 6,23E+06 | 3,12E+06 | 6,23E+05 | 6,23E+05 |
| 9b_2_ | 34,49 | 6,35E+05 | 3,18E+05 | 6,35E+04 | 6,35E+04 |
| 10a_1_ | 28,21 | 5,94E+07 | 2,97E+07 | 5,94E+06 | 5,94E+06 |
| 10a_2_ | 35,06 | 4,21E+05 | 2,10E+05 | 4,21E+04 | 4,21E+04 |
| 10b_1_ | 35,43 | 3,22E+05 | 1,61E+05 | 3,22E+04 | 3,22E+04 |
| 10b_2_ | 36,19 | 1,86E+05 | 9,30E+04 | 1,86E+04 | 0,00E+00 |
| 16a_1_ | 33,2 | 1,61E+06 | 8,07E+05 | 1,61E+05 | 1,61E+05 |
| 16a_2_ | 36,9 | 1,11E+05 | 5,57E+04 | 1,11E+04 | 0,00E+00 |
| 19a | 33,29 | 1,51E+06 | 7,56E+05 | 1,51E+05 | 1,51E+05 |
| 20a_1_ | 32,93 | 1,96E+06 | 9,81E+05 | 1,96E+05 | 1,96E+05 |
| 20a_2_ | 35,17 | 3,89E+05 | 1,94E+05 | 3,89E+04 | 3,89E+04 |
| 20b_1_ | 37,03 | 1,01E+05 | 5,07E+04 | 1,01E+04 | 0,00E+00 |
| 20b_2_ | 34,96 | 4,52E+05 | 2,26E+05 | 4,52E+04 | 4,52E+04 |
| 21a_1_ | 26,79 | 1,66E+08 | 8,28E+07 | 1,66E+07 | 1,66E+07 |
| 21a_2_ | 34,84 | 4,93E+05 | 2,47E+05 | 4,93E+04 | 4,93E+04 |
| 21b_1_ | 36,21 | 1,83E+05 | 9,17E+04 | 1,83E+04 | 0,00E+00 |
| 21b_2_ | 34,03 | 8,86E+05 | 4,43E+05 | 8,86E+04 | 8,86E+04 |
| 21c_1_ | 34,49 | 6,35E+05 | 3,18E+05 | 6,35E+04 | 6,35E+04 |
| 21c_2_ | 34,4 | 6,78E+05 | 3,39E+05 | 6,78E+04 | 6,78E+04 |
| 22a_1_ | 24,78 | 7,08E+08 | 3,54E+08 | 7,08E+07 | 7,08E+07 |
| 22a_2_ | 34,63 | 5,74E+05 | 2,87E+05 | 5,74E+04 | 5,74E+04 |
| 22b_2_ | 33,89 | 9,80E+05 | 4,90E+05 | 9,80E+04 | 9,80E+04 |
| 22b_1_ | 34,51 | 6,26E+05 | 3,13E+05 | 6,26E+04 | 6,26E+04 |
| 23a_1_ | 30,44 | 1,19E+07 | 5,93E+06 | 1,19E+06 | 1,19E+06 |
| 23a_2_ | 34,31 | 7,24E+05 | 3,62E+05 | 7,24E+04 | 7,24E+04 |
| 23b_1_ | 33,69 | 1,13E+06 | 5,66E+05 | 1,13E+05 | 1,13E+05 |
| 23b_2_ | 32,77 | 2,20E+06 | 1,10E+06 | 2,20E+05 | 2,20E+05 |
| 23c_2_ | 34,24 | 7,61E+05 | 3,81E+05 | 7,61E+04 | 7,61E+04 |
| 23d_2_ | 34,27 | 7,45E+05 | 3,72E+05 | 7,45E+04 | 7,45E+04 |
| 24a_1_ | 27,51 | 9,85E+07 | 4,92E+07 | 9,85E+06 | 9,85E+06 |
| 24a_2_ | 25,52 | 4,15E+08 | 2,07E+08 | 4,15E+07 | 4,15E+07 |
| 24b_1_ | 33,27 | 1,53E+06 | 7,67E+05 | 1,53E+05 | 1,53E+05 |
| 24b_2_ | 36,07 | 2,03E+05 | 1,01E+05 | 2,03E+04 | 0,00E+00 |
| 25a_1_ | 22,94 | 2,68E+09 | 1,34E+09 | 2,68E+08 | 2,68E+08 |
| 25a_2_ | 35,91 | 2,28E+05 | 1,14E+05 | 2,28E+04 | 2,28E+04 |
| 25b_1_ | 36,13 | 1,94E+05 | 9,71E+04 | 1,94E+04 | 0,00E+00 |
| 25c_1_ | 34,52 | 6,22E+05 | 3,11E+05 | 6,22E+04 | 6,22E+04 |
| 26a_1_ | 33,44 | 1,36E+06 | 6,78E+05 | 1,36E+05 | 1,36E+05 |
| 26a_2_ | 35,36 | 3,39E+05 | 1,69E+05 | 3,39E+04 | 3,39E+04 |
| 26b_1_ | 33,35 | 1,45E+06 | 7,24E+05 | 1,45E+05 | 1,45E+05 |
| 26b_2_ | 32,35 | 2,98E+06 | 1,49E+06 | 2,98E+05 | 2,98E+05 |
| 27a_1_ | 28,13 | 6,29E+07 | 3,15E+07 | 6,29E+06 | 6,29E+06 |
| 27a_2_ | 35,39 | 3,32E+05 | 1,66E+05 | 3,32E+04 | 3,32E+04 |
| 27b_1_ | 36,72 | 1,27E+05 | 6,34E+04 | 1,27E+04 | 0,00E+00 |
| 27b_2_ | 35,81 | 2,45E+05 | 1,22E+05 | 2,45E+04 | 2,45E+04 |
| 27c_2_ | 36,26 | 1,77E+05 | 8,84E+04 | 1,77E+04 | 0,00E+00 |
| 29a_1_ | 35,44 | 3,20E+05 | 1,60E+05 | 3,20E+04 | 3,20E+04 |
| 29a_2_ | 35,54 | 2,98E+05 | 1,49E+05 | 2,98E+04 | 2,98E+04 |
| 29b_1_ | 35,83 | 2,41E+05 | 1,21E+05 | 2,41E+04 | 2,41E+04 |
| 29b_2_ | 34,86 | 4,86E+05 | 2,43E+05 | 4,86E+04 | 4,86E+04 |

*2.5 Actinomyces naeslundii*

*Standard dilutions and standard curve of A. naeslundii*

| **Log(Copies/reaction)** | **Copy's/reaction** | **Cq** |
| --- | --- | --- |
| 1,02E+01 | 1,65E+10 |  |
| 9,22E+00 | 1,65E+09 | 19,49 |
| 8,22E+00 | 1,65E+08 | 21,28 |
| 7,22E+00 | 1,65E+07 | 24,39 |
| 6,22E+00 | 1,65E+06 | 28,28 |
| 5,22E+00 | 1,65E+05 | 33 ,07 |
| 4,22E+00 | 1,65E+04 |  |
| 3,22E+00 | 1,65E+03 |  |


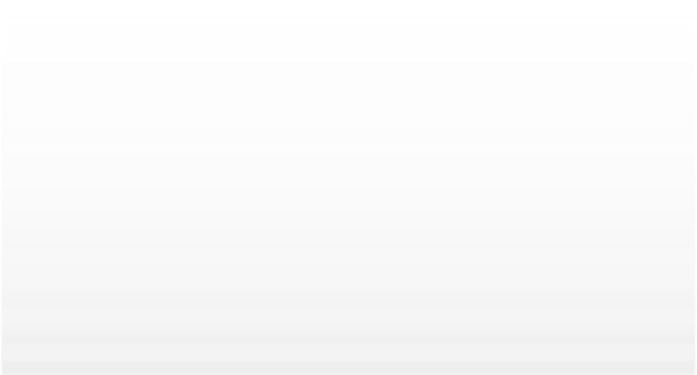

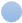

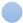

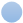

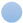

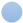


y =

-

49,955

,4156x +

3

R² = 0,9724

00

,

0

,

5

00

00

,

10

00

,

15

20

,

00

25

,

00

00

,

30

00

,

35

00

,

40

00

,

45

1

,

00E+00

00E+00

3

,

5

,

00E+00

7

,

00E+00

9

,

00E+00

1

10E+01

,

^Cq^

Log (copy's/reaction)

Standard curve

*Cq-value, detections in template, number of A. naeslundii in template and number of A. naeslundii/mL*

| **Sample** | Cq | Detections  in  template | Number of MO in template | Number of MO/ml | **Sample** | Cq | Detections  in  template | Number of MO in template | Number of MO/ml |
| --- | --- | --- | --- | --- | --- | --- | --- | --- | --- |
| **AD** | 0,00 | 0,00E+00 | 0,00E+00 | 0,00E+00 | **21b_2_** | 0,00 | 0,00E+00 | 0,00E+00 | 0,00E+00 |
| **AD** | 0,00 | 0,00E+00 | 0,00E+00 | 0,00E+00 | **21c_1_** | 0,00 | 0,00E+00 | 0,00E+00 | 0,00E+00 |
| **AD** | 0,00 | 0,00E+00 | 0,00E+00 | 0,00E+00 | **21c_2_** | 0,00 | 0,00E+00 | 0,00E+00 | 0,00E+00 |
| **1** | 31,15 | 1,60E+03 | 1,60E+03 | 6,39E+04 | **22a_1_** | 0,00 | 0,00E+00 | 0,00E+00 | 0,00E+00 |
| **2** | 0,00 | 0,00E+00 | 0,00E+00 | 0,00E+00 | **22a_2_** | 0,00 | 0,00E+00 | 0,00E+00 | 0,00E+00 |
| **3a_1_** | 0,00 | 0,00E+00 | 0,00E+00 | 0,00E+00 | **22b_1_** | 0,00 | 0,00E+00 | 0,00E+00 | 0,00E+00 |
| **3a_2_** | 0,00 | 0,00E+00 | 0,00E+00 | 0,00E+00 | **22b_2_** | 0,00 | 0,00E+00 | 0,00E+00 | 0,00E+00 |
| **3b_1_** | 0,00 | 0,00E+00 | 0,00E+00 | 0,00E+00 | **23a_1_** | 0,00 | 0,00E+00 | 0,00E+00 | 0,00E+00 |
| **3b_2_** | 0,00 | 0,00E+00 | 0,00E+00 | 0,00E+00 | **23a_2_** | 0,00 | 0,00E+00 | 0,00E+00 | 0,00E+00 |
| **4a_1_** | 0,00 | 0,00E+00 | 0,00E+00 | 0,00E+00 | **23b_1_** | 0,00 | 0,00E+00 | 0,00E+00 | 0,00E+00 |
| **4a_2_** | 0,00 | 0,00E+00 | 0,00E+00 | 0,00E+00 | **23b_2_** | 0,00 | 0,00E+00 | 0,00E+00 | 0,00E+00 |
| **4b_1_** | 0,00 | 0,00E+00 | 0,00E+00 | 0,00E+00 | **23c_2_** | 0,00 | 0,00E+00 | 0,00E+00 | 0,00E+00 |
| **4b_2_** | 0,00 | 0,00E+00 | 0,00E+00 | 0,00E+00 | **23d_2_** | 0,00 | 0,00E+00 | 0,00E+00 | 0,00E+00 |
| **5a_1_** | 0,00 | 0,00E+00 | 0,00E+00 | 0,00E+00 | **24a_1_** | 0,00 | 0,00E+00 | 0,00E+00 | 0,00E+00 |
| **5a_2_** | 0,00 | 0,00E+00 | 0,00E+00 | 0,00E+00 | **24a_2_** | 0,00 | 0,00E+00 | 0,00E+00 | 0,00E+00 |
| **5b_1_** | 0,00 | 0,00E+00 | 0,00E+00 | 0,00E+00 | **24b_1_** | 0,00 | 0,00E+00 | 0,00E+00 | 0,00E+00 |
| **5b_2_** | 0,00 | 0,00E+00 | 0,00E+00 | 0,00E+00 | **24b_2_** | 0,00 | 0,00E+00 | 0,00E+00 | 0,00E+00 |
| **9a_1_** | 0,00 | 0,00E+00 | 0,00E+00 | 0,00E+00 | **25a_1_** | 0,00 | 0,00E+00 | 0,00E+00 | 0,00E+00 |
| **9a_2_** | 0,00 | 0,00E+00 | 0,00E+00 | 0,00E+00 | **25a_2_** | 0,00 | 0,00E+00 | 0,00E+00 | 0,00E+00 |
| **9b_1_** | 0,00 | 0,00E+00 | 0,00E+00 | 0,00E+00 | **25b_1_** | 0,00 | 0,00E+00 | 0,00E+00 | 0,00E+00 |
| **9b_2_** | 0,00 | 0,00E+00 | 0,00E+00 | 0,00E+00 | **25c_1_** | 0,00 | 0,00E+00 | 0,00E+00 | 0,00E+00 |
| **10a_1_** | 0,00 | 0,00E+00 | 0,00E+00 | 0,00E+00 | **26a_1_** | 0,00 | 0,00E+00 | 0,00E+00 | 0,00E+00 |
| **10a_2_** | 36,83 | 3,48E+01 | 3,48E+01 | 1,39E+03 | **26a_2_** | 0,00 | 0,00E+00 | 0,00E+00 | 0,00E+00 |
| **10b_1_** | 0,00 | 0,00E+00 | 0,00E+00 | 0,00E+00 | **26b_1_** | 0,00 | 0,00E+00 | 0,00E+00 | 0,00E+00 |
| **10b_2_** | 37,19 | 2,72E+01 | 2,72E+01 | 1,09E+03 | **26b_2_** | 0,00 | 0,00E+00 | 0,00E+00 | 0,00E+00 |
| **16_1_** | 0,00 | 0,00E+00 | 0,00E+00 | 0,00E+00 | **27a_1_** | 0,00 | 0,00E+00 | 0,00E+00 | 0,00E+00 |
| **16_2_** | 0,00 | 0,00E+00 | 0,00E+00 | 0,00E+00 | **27a_2_** | 0,00 | 0,00E+00 | 0,00E+00 | 0,00E+00 |
| **19** | 0,00 | 0,00E+00 | 0,00E+00 | 0,00E+00 | **27b_1_** | 0,00 | 0,00E+00 | 0,00E+00 | 0,00E+00 |
| **20a_1_** | 0,00 | 0,00E+00 | 0,00E+00 | 0,00E+00 | **27b_2_** | 0,00 | 0,00E+00 | 0,00E+00 | 0,00E+00 |
| **20a_2_** | 0,00 | 0,00E+00 | 0,00E+00 | 0,00E+00 | **27c_2_** | 0,00 | 0,00E+00 | 0,00E+00 | 0,00E+00 |
| **20b_1_** | 40,34 | 3,27E+00 | 3,27E+00 | 1,31E+02 | **29a_1_** | 0,00 | 0,00E+00 | 0,00E+00 | 0,00E+00 |
| **20b_2_** | 0,00 | 0,00E+00 | 0,00E+00 | 0,00E+00 | **29a_2_** | 0,00 | 0,00E+00 | 0,00E+00 | 0,00E+00 |
| **21a_1_** | 0,00 | 0,00E+00 | 0,00E+00 | 0,00E+00 | **29b_1_** | 0,00 | 0,00E+00 | 0,00E+00 | 0,00E+00 |
| **21a_2_** | 0,00 | 0,00E+00 | 0,00E+00 | 0,00E+00 | **29b_2_** | 0,00 | 0,00E+00 | 0,00E+00 | 0,00E+00 |
| **21b_1_** | 0,00 | 0,00E+00 | 0,00E+00 | 0,00E+00 |  |  |  |  |  |

*2.6 Filifactor alocis*

*Standard dilutions and standard curve of F. alocis*

| **Log(Copy's/reaction)** | **Copy's/reaction** | **Cq** |
| --- | --- | --- |
| 1,02E+01 | 1,65E+10 | 13,49 |
| 9,22E+00 | 1,65E+09 | 17,45 |
| 8,22E+00 | 1,65E+08 | 21,12 |
| 7,22E+00 | 1,65E+07 | 24,46 |
| 6,22E+00 | 1,65E+06 | 27,58 |
| 5,22E+00 | 1,65E+05 | 30,70 |
| 4,22E+00 | 1,65E+04 | 34 ,02 |
| 3,22E+00 | 1,65E+03 |  |


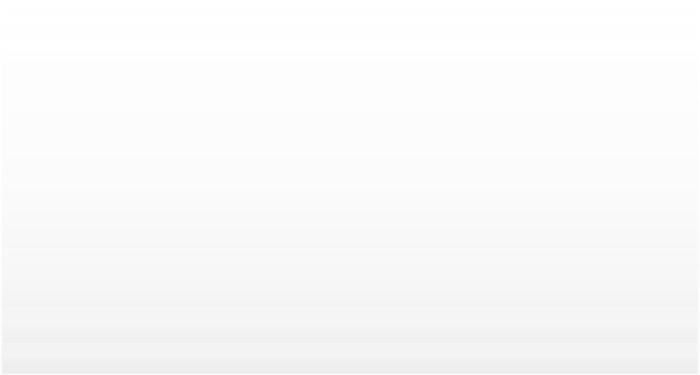

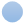

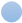

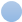

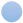

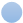

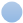

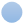


y =

-

3

,3757x +

48,484

R² = 0,9981

0

00

,

00

,

5

00

,

10

00

,

15

00

20

,

00

,

25

30

,

00

00

,

35

40

00

,

1

,

00E+00

,

3

00E+00

5

,

00E+00

00E+00

7

,

9

,

00E+00

10E+01

,

1

^Cq^

Log (copy's/reaction)

Standard curve

*Cq-value, detections in template, number of F. alocis in template and number of F. alocis/mL*

| **Sample** | Cq | Detections  in  template | Number of MO in template | Number of MO/ml | **Sample** | Cq | Detections  in  template | Number of MO in template | Number of MO/ml |
| --- | --- | --- | --- | --- | --- | --- | --- | --- | --- |
| **AD** | 0,00 | 0,00E+00 | 0,00E+00 | 0,00E+00 | **21b_1_** | 0,00 | 0,00E+00 | 0,00E+00 | 0,00E+00 |
| **AD** | 0,00 | 0,00E+00 | 0,00E+00 | 0,00E+00 | **21b_2_** | 0,00 | 0,00E+00 | 0,00E+00 | 0,00E+00 |
| **AD** | 0,00 | 0,00E+00 | 0,00E+00 | 0,00E+00 | **21c_1_** | 0,00 | 0,00E+00 | 0,00E+00 | 0,00E+00 |
| **AD** | 0,00 | 0,00E+00 | 0,00E+00 | 0,00E+00 | **21c_2_** | 0,00 | 0,00E+00 | 0,00E+00 | 0,00E+00 |
| **1** | 0,00 | 0,00E+00 | 0,00E+00 | 0,00E+00 | **22a_1_** | 0,00 | 0,00E+00 | 0,00E+00 | 0,00E+00 |
| **2** | 0,00 | 0,00E+00 | 0,00E+00 | 0,00E+00 | **22a_2_** | 0,00 | 0,00E+00 | 0,00E+00 | 0,00E+00 |
| **3a_1_** | 0,00 | 0,00E+00 | 0,00E+00 | 0,00E+00 | **22b_1_** | 0,00 | 0,00E+00 | 0,00E+00 | 0,00E+00 |
| **3a_2_** | 0,00 | 0,00E+00 | 0,00E+00 | 0,00E+00 | **22b_2_** | 0,00 | 0,00E+00 | 0,00E+00 | 0,00E+00 |
| **3b_1_** | 0,00 | 0,00E+00 | 0,00E+00 | 0,00E+00 | **23a_1_** | 0,00 | 0,00E+00 | 0,00E+00 | 0,00E+00 |
| **3b_2_** | 0,00 | 0,00E+00 | 0,00E+00 | 0,00E+00 | **23a_2_** | 0,00 | 0,00E+00 | 0,00E+00 | 0,00E+00 |
| **4a_1_** | 0,00 | 0,00E+00 | 0,00E+00 | 0,00E+00 | **23b_1_** | 0,00 | 0,00E+00 | 0,00E+00 | 0,00E+00 |
| **4a_2_** | 0,00 | 0,00E+00 | 0,00E+00 | 0,00E+00 | **23b_2_** | 0,00 | 0,00E+00 | 0,00E+00 | 0,00E+00 |
| **4b_1_** | 0,00 | 0,00E+00 | 0,00E+00 | 0,00E+00 | **23c_2_** | 0,00 | 0,00E+00 | 0,00E+00 | 0,00E+00 |
| **4b_2_** | 0,00 | 0,00E+00 | 0,00E+00 | 0,00E+00 | **23d_2_** | 0,00 | 0,00E+00 | 0,00E+00 | 0,00E+00 |
| **5a_1_** | 0,00 | 0,00E+00 | 0,00E+00 | 0,00E+00 | **24a_1_** | 31,01 | 7,51E+02 | 7,51E+02 | 3,00E+04 |
| **5a_2_** | 0,00 | 0,00E+00 | 0,00E+00 | 0,00E+00 | **24a_2_** | 28,16 | 5,23E+03 | 5,23E+03 | 2,09E+05 |
| **5b_1_** | 0,00 | 0,00E+00 | 0,00E+00 | 0,00E+00 | **24b_1_** | 37,19 | 1,11E+01 | 1,11E+01 | 4,43E+02 |
| **5b_2_** | 0,00 | 0,00E+00 | 0,00E+00 | 0,00E+00 | **24b_2_** | 0,00 | 0,00E+00 | 0,00E+00 | 0,00E+00 |
| **9a_1_** | 0,00 | 0,00E+00 | 0,00E+00 | 0,00E+00 | **25a_1_** | 0,00 | 0,00E+00 | 0,00E+00 | 0,00E+00 |
| **9a_2_** | 0,00 | 0,00E+00 | 0,00E+00 | 0,00E+00 | **25a_2_** | 0,00 | 0,00E+00 | 0,00E+00 | 0,00E+00 |
| **9b_1_** | 0,00 | 0,00E+00 | 0,00E+00 | 0,00E+00 | **25b_1_** | 0,00 | 0,00E+00 | 0,00E+00 | 0,00E+00 |
| **9b_2_** | 0,00 | 0,00E+00 | 0,00E+00 | 0,00E+00 | **25c_1_** | 0,00 | 0,00E+00 | 0,00E+00 | 0,00E+00 |
| **10a_1_** | 0,00 | 0,00E+00 | 0,00E+00 | 0,00E+00 | **26a_1_** | 0,00 | 0,00E+00 | 0,00E+00 | 0,00E+00 |
| **10a_2_** | 0,00 | 0,00E+00 | 0,00E+00 | 0,00E+00 | **26a_2_** | 0,00 | 0,00E+00 | 0,00E+00 | 0,00E+00 |
| **10b_1_** | 0,00 | 0,00E+00 | 0,00E+00 | 0,00E+00 | **26b_1_** | 0,00 | 0,00E+00 | 0,00E+00 | 0,00E+00 |
| **10b_2_** | 0,00 | 0,00E+00 | 0,00E+00 | 0,00E+00 | **26b_2_** | 0,00 | 0,00E+00 | 0,00E+00 | 0,00E+00 |
| **16_1_** | 0,00 | 0,00E+00 | 0,00E+00 | 0,00E+00 | **27a_1_** | 0,00 | 1,15E+12 | 1,15E+12 | 4,61E+13 |
| **16_2_** | 0,00 | 0,00E+00 | 0,00E+00 | 0,00E+00 | **27a_2_** | 0,00 | 0,00E+00 | 0,00E+00 | 0,00E+00 |
| **19** | 0,00 | 0,00E+00 | 0,00E+00 | 0,00E+00 | **27b_1_** | 0,00 | 0,00E+00 | 0,00E+00 | 0,00E+00 |
| **20a_1_** | 0,00 | 0,00E+00 | 0,00E+00 | 0,00E+00 | **27b_2_** | 0,00 | 0,00E+00 | 0,00E+00 | 0,00E+00 |
| **20a_2_** | 0,00 | 0,00E+00 | 0,00E+00 | 0,00E+00 | **27c_2_** | 36,13 | 2,29E+01 | 2,29E+01 | 9,14E+02 |
| **20b_1_** | 0,00 | 0,00E+00 | 0,00E+00 | 0,00E+00 | **29a_1_** | 0,00 | 0,00E+00 | 0,00E+00 | 0,00E+00 |
| **20b_2_** | 0,00 | 0,00E+00 | 0,00E+00 | 0,00E+00 | **29a_2_** | 0,00 | 0,00E+00 | 0,00E+00 | 0,00E+00 |
| **21a_1_** | 0,00 | 0,00E+00 | 0,00E+00 | 0,00E+00 | **29b_1_** | 0,00 | 0,00E+00 | 0,00E+00 | 0,00E+00 |
| **21a_2_** | 0,00 | 0,00E+00 | 0,00E+00 | 0,00E+00 | **29b_2_** | 0,00 | 0,00E+00 | 0,00E+00 | 0,00E+00 |

### *2.7 Fusobacterium nucleatum*

#### Standard dilutions and standard curve of F. nucleatum

| **Log(Copy's/reaction)** | **Copy's/reaction** | **Cq** |
| --- | --- | --- |
| 1,02E+01 | 1,62E+10 | 13,38 |
| 9,21E+00 | 1,62E+09 | 17,18 |
| 8,21E+00 | 1,62E+08 | 19,42 |
| 7,21E+00 | 1,62E+07 | 23,11 |
| 6,21E+00 | 1,62E+06 | 27,47 |
| 5,21E+00 | 1,62E+05 | 31 ,59 |
| 4,21E+00 | 1,62E+04 |  |
| 3,21E+00 | 1,62E+03 |  |


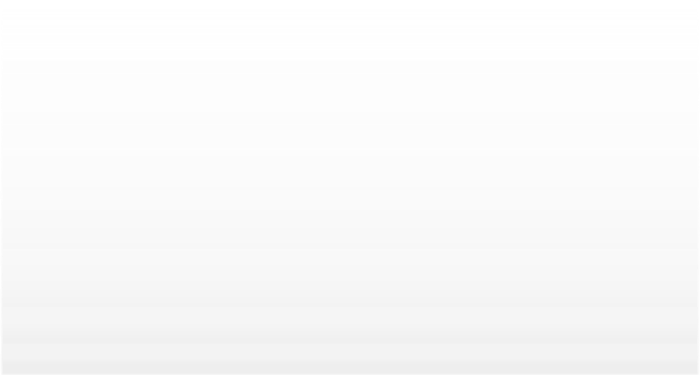

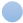

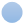

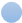

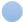

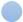

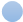


y =

-

3

49,698

,5895x +

R² = 0,9916

00

0

,

5

,

00

00

,

10

15

,

00

20

,

00

,

25

00

30

,

00

00

35

,

00

40

,

00

,

45

1

,

00E+00

3

,

00E+00

5

,

00E+00

7

,

00E+00

9

00E+00

,

1

,

10E+01

^Cq^

Log (copy's/reaction)

Standard curve

### *Cq-value, detections in template, number of F. nucleatum in template and number of F. nucleatum/mL*

| **Sample** | Cq | Detections in template | Number of  MO in  template | Number of MO/ml | **Sample** | Cq | Detections  in  template | Number of MO in template | Number of MO/ml |
| --- | --- | --- | --- | --- | --- | --- | --- | --- | --- |
| **AD** | 0,00 | 0,00E+00 | 0,00E+00 | 0,00E+00 | **21b_2_** | 34,81 | 7,01E+01 | 1,40E+01 | 5,61E+02 |
| **AD** | 0,00 | 0,00E+00 | 0,00E+00 | 0,00E+00 | **21c_1_** | 38,16 | 8,18E+00 | 1,64E+00 | 6,54E+01 |
| **AD** | 0,00 | 0,00E+00 | 0,00E+00 | 0,00E+00 | **21c_2_** | 33,49 | 1,64E+02 | 3,28E+01 | 1,31E+03 |
| **1** | 17,23 | 5,57E+06 | 1,11E+06 | 4,45E+07 | **22a_1_** | 19,88 | 1,02E+06 | 2,03E+05 | 8,13E+06 |
| **2** | 32,55 | 2,99E+02 | 5,98E+01 | 2,39E+03 | **22a_2_** | 36,55 | 2,30E+01 | 4,61E+00 | 1,84E+02 |
| **3a_1_** | 21,12 | 4,59E+05 | 9,18E+04 | 3,67E+06 | **22b_1_** | 35,58 | 4,28E+01 | 8,56E+00 | 3,43E+02 |
| **3a_2_** | 29,25 | 2,48E+03 | 4,97E+02 | 1,99E+04 | **22b_2_** | 30,17 | 1,38E+03 | 2,76E+02 | 1,10E+04 |
| **3b_1_** | 31,99 | 4,30E+02 | 8,60E+01 | 3,44E+03 | **23a_1_** | 21,87 | 2,84E+05 | 5,67E+04 | 2,27E+06 |
| **3b_2_** | 36,80 | 1,95E+01 | 3,91E+00 | 1,56E+02 | **23a_2_** | 32,98 | 2,27E+02 | 4,53E+01 | 1,81E+03 |
| **4a_1_** | 22,02 | 2,56E+05 | 5,12E+04 | 2,05E+06 | **23b_1_** | 35,40 | 4,81E+01 | 9,62E+00 | 3,85E+02 |
| **4a_2_** | 34,27 | 9,91E+01 | 1,98E+01 | 7,93E+02 | **23b_2_** | 31,37 | 6,38E+02 | 1,28E+02 | 5,10E+03 |
| **4b_1_** | 35,90 | 3,49E+01 | 6,99E+00 | 2,80E+02 | **23c_2_** | 36,85 | 1,89E+01 | 3,79E+00 | 1,52E+02 |
| **4b_2_** | 0,00 | 0,00E+00 | 0,00E+00 | 0,00E+00 | **23d_2_** | 33,38 | 1,75E+02 | 3,51E+01 | 1,40E+03 |
| **5a_1_** | 25,59 | 2,61E+04 | 5,22E+03 | 2,09E+05 | **24a_1_** | 28,48 | 4,07E+03 | 8,14E+02 | 3,26E+04 |
| **5a_2_** | 36,18 | 2,91E+01 | 5,82E+00 | 2,33E+02 | **24a_2_** | 25,63 | 2,54E+04 | 5,08E+03 | 2,03E+05 |
| **5b_1_** | 34,43 | 8,94E+01 | 1,79E+01 | 7,15E+02 | **24b_1_** | 34,16 | 1,07E+02 | 2,13E+01 | 8,52E+02 |
| **5b_2_** | 38,09 | 8,56E+00 | 1,71E+00 | 6,85E+01 | **24b_2_** | 38,12 | 8,43E+00 | 1,69E+00 | 6,74E+01 |
| **9a_1_** | 31,54 | 5,72E+02 | 1,14E+02 | 4,58E+03 | **25a_1_** | 23,59 | 9,36E+04 | 1,87E+04 | 7,49E+05 |
| **9a_2_** | 0,00 | 0,00E+00 | 0,00E+00 | 0,00E+00 | **25a_2_** | 0,00 | 0,00E+00 | 0,00E+00 | 0,00E+00 |
| **9b_1_** | 32,70 | 2,73E+02 | 5,45E+01 | 2,18E+03 | **25b_1_** | 37,31 | 1,42E+01 | 2,83E+00 | 1,13E+02 |
| **9b_2_** | 38,17 | 8,11E+00 | 1,62E+00 | 6,49E+01 | **25c_1_** | 33,02 | 2,22E+02 | 4,44E+01 | 1,77E+03 |
| **10a_1_** | 26,40 | 1,55E+04 | 3,09E+03 | 1,24E+05 | **26a_1_** | 25,97 | 2,04E+04 | 4,07E+03 | 1,63E+05 |
| **10a_2_** | 0,00 | 0,00E+00 | 0,00E+00 | 0,00E+00 | **26a_2_** | 35,53 | 4,42E+01 | 8,83E+00 | 3,53E+02 |
| **10b_1_** | 33,60 | 1,53E+02 | 3,06E+01 | 1,22E+03 | **26b_1_** | 36,58 | 2,25E+01 | 4,51E+00 | 1,80E+02 |
| **10b_2_** | 38,98 | 4,85E+00 | 9,69E-01 | 3,88E+01 | **26b_2_** | 30,11 | 1,43E+03 | 2,87E+02 | 1,15E+04 |
| **16_1_** | 35,60 | 4,23E+01 | 8,46E+00 | 3,38E+02 | **27a_1_** | 28,13 | 5,10E+03 | 1,02E+03 | 4,08E+04 |
| **16_2_** | 33,31 | 1,84E+02 | 3,68E+01 | 1,47E+03 | **27a_2_** | 39,50 | 3,48E+00 | 6,95E-01 | 2,78E+01 |
| **19** | 31,84 | 4,72E+02 | 9,45E+01 | 3,78E+03 | **27b_1_** | 36,94 | 1,80E+01 | 3,59E+00 | 1,44E+02 |
| **20a_1_** | 21,85 | 2,86E+05 | 5,72E+04 | 2,29E+06 | **27b_2_** | 37,31 | 1,42E+01 | 2,83E+00 | 1,13E+02 |
| **20a_2_** | 37,51 | 1,24E+01 | 2,48E+00 | 9,94E+01 | **27c_2_** | 36,38 | 2,57E+01 | 5,14E+00 | 2,06E+02 |
| **20b_1_** | 37,37 | 1,36E+01 | 2,72E+00 | 1,09E+02 | **29a_1_** | 0,00 | 0,00E+00 | 0,00E+00 | 0,00E+00 |
| **20b_2_** | 38,24 | 7,77E+00 | 1,55E+00 | 6,21E+01 | **29a_2_** | 34,07 | 1,13E+02 | 2,25E+01 | 9,01E+02 |
| **21a_1_** | 24,05 | 6,98E+04 | 1,40E+04 | 5,58E+05 | **29b_1_** | 35,44 | 4,68E+01 | 9,35E+00 | 3,74E+02 |
| **21a_2_** | 34,96 | 6,40E+01 | 1,28E+01 | 5,12E+02 | **29b_2_** | 36,17 | 2,94E+01 | 5,88E+00 | 2,35E+02 |
| **21b_1_** | 0,00 | 0,00E+00 | 0,00E+00 | 0,00E+00 |  |  |  |  |  |

### *2.8 Porphyromonas gingivalis*

### *Standard dilutions and standard curve of P. gingivalis*

| **Log(Copy's/reaction)** | **Copy's/reaction** | **Cq** |
| --- | --- | --- |
| 1,03E+01 | 2,12E+10 | 15,98 |
| 9,33E+00 | 2,12E+09 | 19,55 |
| 8,33E+00 | 2,12E+08 | 23,27 |
| 7,33E+00 | 2,12E+07 | 25,99 |
| 6,33E+00 | 2,12E+06 | 30,32 |
| 5,33E+00 | 2,12E+05 | 33 ,04 |
| 4,33E+00 | 2,12E+04 |  |
| 3,33E+00 | 2,12E+03 |  |


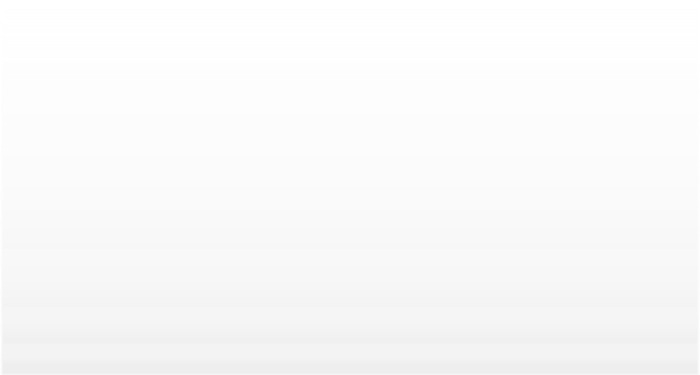

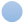

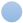

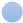

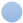

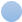

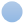


y =

-

3

51,602

,4382x +

R² = 0,9973

00

0

,

5

,

00

00

,

10

15

,

00

20

,

00

,

25

00

30

,

00

00

35

,

00

40

,

00

,

45

1

,

00E+00

3

,

00E+00

5

,

00E+00

7

,

00E+00

9

00E+00

,

1

,

10E+01

^Cq^

Log (copy's/reactie)

Standard curve

### *Cq-value, detections in template, number of P. gingivalis in template and number of P. gingivalis/mL*

| **Sample** | Cq | Detections in template | Number of  MO in  template | Number of MO/ml | **Sample** | Cq | Detections  in  template | Number of MO in template | Number of MO/ml |
| --- | --- | --- | --- | --- | --- | --- | --- | --- | --- |
| **AD** | 0,00 | 0,00E+00 | 0,00E+00 | 0,00E+00 | **21b_1_** | 0,00 | 0,00E+00 | 0,00E+00 | 0,00E+00 |
| **AD** | 0,00 | 0,00E+00 | 0,00E+00 | 0,00E+00 | **21b_2_** | 0,00 | 0,00E+00 | 0,00E+00 | 0,00E+00 |
| **AD** | 0,00 | 0,00E+00 | 0,00E+00 | 0,00E+00 | **21c_1_** | 0,00 | 0,00E+00 | 0,00E+00 | 0,00E+00 |
| **AD** | 0,00 | 0,00E+00 | 0,00E+00 | 0,00E+00 | **21c_2_** | 0,00 | 0,00E+00 | 0,00E+00 | 0,00E+00 |
| **1** | 19,77 | 9,05E+06 | 2,26E+06 | 1,95E+08 | **22a_1_** | 0,00 | 0,00E+00 | 0,00E+00 | 0,00E+00 |
| **2** | 0,00 | 0,00E+00 | 0,00E+00 | 0,00E+00 | **22a_2_** | 0,00 | 0,00E+00 | 0,00E+00 | 0,00E+00 |
| **3a_1_** | 0,00 | 0,00E+00 | 0,00E+00 | 0,00E+00 | **22b_1_** | 0,00 | 0,00E+00 | 0,00E+00 | 0,00E+00 |
| **3a_2_** | 0,00 | 0,00E+00 | 0,00E+00 | 0,00E+00 | **22b_2_** | 0,00 | 0,00E+00 | 0,00E+00 | 0,00E+00 |
| **3b_1_** | 0,00 | 0,00E+00 | 0,00E+00 | 0,00E+00 | **23a_1_** | 0,00 | 0,00E+00 | 0,00E+00 | 0,00E+00 |
| **3b_2_** | 0,00 | 0,00E+00 | 0,00E+00 | 0,00E+00 | **23a_2_** | 0,00 | 0,00E+00 | 0,00E+00 | 0,00E+00 |
| **4a_1_** | 0,00 | 0,00E+00 | 0,00E+00 | 0,00E+00 | **23b_1_** | 0,00 | 0,00E+00 | 0,00E+00 | 0,00E+00 |
| **4a_2_** | 0,00 | 0,00E+00 | 0,00E+00 | 0,00E+00 | **23b_2_** | 0,00 | 0,00E+00 | 0,00E+00 | 0,00E+00 |
| **4b_1_** | 41,67 | 3,87E+00 | 9,67E-01 | 8,36E+01 | **23c_2_** | 0,00 | 0,00E+00 | 0,00E+00 | 0,00E+00 |
| **4b_2_** | 0,00 | 0,00E+00 | 0,00E+00 | 0,00E+00 | **23d_2_** | 0,00 | 0,00E+00 | 0,00E+00 | 0,00E+00 |
| **5a_1_** | 0,00 | 0,00E+00 | 0,00E+00 | 0,00E+00 | **24a_1_** | 0,00 | 0,00E+00 | 0,00E+00 | 0,00E+00 |
| **5a_2_** | 0,00 | 0,00E+00 | 0,00E+00 | 0,00E+00 | **24a_2_** | 0,00 | 0,00E+00 | 0,00E+00 | 0,00E+00 |
| **5b_1_** | 0,00 | 0,00E+00 | 0,00E+00 | 0,00E+00 | **24b_1_** | 0,00 | 0,00E+00 | 0,00E+00 | 0,00E+00 |
| **5b_2_** | 0,00 | 0,00E+00 | 0,00E+00 | 0,00E+00 | **24b_2_** | 0,00 | 0,00E+00 | 0,00E+00 | 0,00E+00 |
| **9a_1_** | 0,00 | 0,00E+00 | 0,00E+00 | 0,00E+00 | **25a_1_** | 0,00 | 0,00E+00 | 0,00E+00 | 0,00E+00 |
| **9a_2_** | 0,00 | 0,00E+00 | 0,00E+00 | 0,00E+00 | **25a_2_** | 0,00 | 0,00E+00 | 0,00E+00 | 0,00E+00 |
| **9b_1_** | 0,00 | 0,00E+00 | 0,00E+00 | 0,00E+00 | **25b_1_** | 0,00 | 0,00E+00 | 0,00E+00 | 0,00E+00 |
| **9b_2_** | 0,00 | 0,00E+00 | 0,00E+00 | 0,00E+00 | **25c_1_** | 0,00 | 0,00E+00 | 0,00E+00 | 0,00E+00 |
| **10a_1_** | 0,00 | 0,00E+00 | 0,00E+00 | 0,00E+00 | **26a_1_** | 0,00 | 0,00E+00 | 0,00E+00 | 0,00E+00 |
| **10a_2_** | 0,00 | 0,00E+00 | 0,00E+00 | 0,00E+00 | **26a_2_** | 0,00 | 0,00E+00 | 0,00E+00 | 0,00E+00 |
| **10b_1_** | 0,00 | 0,00E+00 | 0,00E+00 | 0,00E+00 | **26b_1_** | 0,00 | 0,00E+00 | 0,00E+00 | 0,00E+00 |
| **10b_2_** | 0,00 | 0,00E+00 | 0,00E+00 | 0,00E+00 | **26b_2_** | 0,00 | 0,00E+00 | 0,00E+00 | 0,00E+00 |
| **16_1_** | 0,00 | 0,00E+00 | 0,00E+00 | 0,00E+00 | **27a_1_** | 0,00 | 0,00E+00 | 0,00E+00 | 0,00E+00 |
| **16_2_** | 0,00 | 0,00E+00 | 0,00E+00 | 0,00E+00 | **27a_2_** | 0,00 | 0,00E+00 | 0,00E+00 | 0,00E+00 |
| **19** | 0,00 | 0,00E+00 | 0,00E+00 | 0,00E+00 | **27b_1_** | 0,00 | 0,00E+00 | 0,00E+00 | 0,00E+00 |
| **20a_1_** | 0,00 | 0,00E+00 | 0,00E+00 | 0,00E+00 | **27b_2_** | 0,00 | 0,00E+00 | 0,00E+00 | 0,00E+00 |
| **20a_2_** | 0,00 | 0,00E+00 | 0,00E+00 | 0,00E+00 | **27c_2_** | 0,00 | 0,00E+00 | 0,00E+00 | 0,00E+00 |
| **20b_1_** | 0,00 | 0,00E+00 | 0,00E+00 | 0,00E+00 | **29a_1_** | 0,00 | 0,00E+00 | 0,00E+00 | 0,00E+00 |
| **20b_2_** | 0,00 | 0,00E+00 | 0,00E+00 | 0,00E+00 | **29a_2_** | 0,00 | 0,00E+00 | 0,00E+00 | 0,00E+00 |
| **21a_1_** | 0,00 | 0,00E+00 | 0,00E+00 | 0,00E+00 | **29b_1_** | 0,00 | 0,00E+00 | 0,00E+00 | 0,00E+00 |
| **21a_2_** | 0,00 | 0,00E+00 | 0,00E+00 | 0,00E+00 | **29b_2_** | 0,00 | 0,00E+00 | 0,00E+00 | 0,00E+00 |

### *2.9 Prevotella intermedia*

### *Standard dilutions and standard curve of P. intermedia*

| **Log(Copy's/reaction)** | **Copy's/reaction** | **Ct** |
| --- | --- | --- |
| 1,01E+01 | 1,26E+10 | 15,27 |
| 9,10E+00 | 1,26E+09 | 18,33 |
| 8,10E+00 | 1,26E+08 | 21,56 |
| 7,10E+00 | 1,26E+07 | 24,76 |
| 6,10E+00 | 1,26E+06 | 27,64 |
| 5,10E+00 | 1,26E+05 | 32,41 |
| 4,10E+00 | 1,26E+04 | 34 ,54 |
| 3,10E+00 | 1,26E+03 |  |


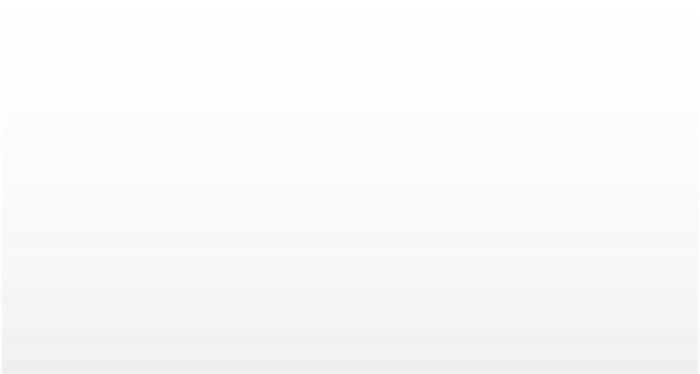

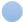

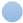

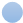

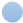

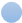

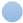

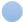


y =

-

48,271

,2873x +

3

R² = 0,9957

0

,

00

,

00

5

,

10

00

,

15

00

20

,

00

,

00

25

,

30

00

00

,

35

40

,

00

1

,

00E+00

3

,

00E+00

5

,

00E+00

,

00E+00

7

9

00E+00

,

1

,

10E+01

^Cq^

Log (copy's/reaction)

Standard curve

### *Cq-value, detections in template, number of P. intermedia in template and number of P. intermedia/mL*

| **Sample** | Cq | Detections in template | Number of  MO in  template | Number of MO/ml | **Sample** | Cq | Detections  in  template | Number of MO in template | Number of MO/ml |
| --- | --- | --- | --- | --- | --- | --- | --- | --- | --- |
| **AD** | 0,00 | 0,00E+00 | 0,00E+00 | 0,00E+00 | **21b_1_** | 0,00 | 0,00E+00 | 0,00E+00 | 0,00E+00 |
| **AD** | 0,00 | 0,00E+00 | 0,00E+00 | 0,00E+00 | **21b_2_** | 0,00 | 0,00E+00 | 0,00E+00 | 0,00E+00 |
| **AD** | 0,00 | 0,00E+00 | 0,00E+00 | 0,00E+00 | **21c_1_** | 0,00 | 0,00E+00 | 0,00E+00 | 0,00E+00 |
| **AD** | 0,00 | 0,00E+00 | 0,00E+00 | 0,00E+00 | **21c_2_** | 0,00 | 0,00E+00 | 0,00E+00 | 0,00E+00 |
| **1** | 21,87 | 5,39E+05 | 1,80E+05 | 7,61E+06 | **22a_1_** | 37,29 | 1,10E+01 | 3,65E+00 | 1,55E+02 |
| **2** | 0,00 | 0,00E+00 | 0,00E+00 | 0,00E+00 | **22a_2_** | 35,17 | 4,82E+01 | 1,61E+01 | 6,82E+02 |
| **3a_1_** | 0,00 | 0,00E+00 | 0,00E+00 | 0,00E+00 | **22b_1_** | 0,00 | 0,00E+00 | 0,00E+00 | 0,00E+00 |
| **3a_2_** | 0,00 | 0,00E+00 | 0,00E+00 | 0,00E+00 | **22b_2_** | 0,00 | 0,00E+00 | 0,00E+00 | 0,00E+00 |
| **3b_1_** | 0,00 | 0,00E+00 | 0,00E+00 | 0,00E+00 | **23a_1_** | 0,00 | 0,00E+00 | 0,00E+00 | 0,00E+00 |
| **3b_2_** | 0,00 | 0,00E+00 | 0,00E+00 | 0,00E+00 | **23a_2_** | 0,00 | 0,00E+00 | 0,00E+00 | 0,00E+00 |
| **4a_1_** | 0,00 | 0,00E+00 | 0,00E+00 | 0,00E+00 | **23b_1_** | 0,00 | 0,00E+00 | 0,00E+00 | 0,00E+00 |
| **4a_2_** | 0,00 | 0,00E+00 | 0,00E+00 | 0,00E+00 | **23b_2_** | 0,00 | 0,00E+00 | 0,00E+00 | 0,00E+00 |
| **4b_1_** | 0,00 | 0,00E+00 | 0,00E+00 | 0,00E+00 | **23c_2_** | 0,00 | 0,00E+00 | 0,00E+00 | 0,00E+00 |
| **4b_2_** | 0,00 | 0,00E+00 | 0,00E+00 | 0,00E+00 | **23d_2_** | 37,17 | 1,19E+01 | 3,97E+00 | 1,69E+02 |
| **5a_1_** | 28,99 | 3,66E+03 | 1,22E+03 | 5,17E+04 | **24a_1_** | 0,00 | 0,00E+00 | 0,00E+00 | 0,00E+00 |
| **5a_2_** | 0,00 | 0,00E+00 | 0,00E+00 | 0,00E+00 | **24a_2_** | 0,00 | 2,42E+12 | 8,05E+11 | 3,41E+13 |
| **5b_1_** | 0,00 | 0,00E+00 | 0,00E+00 | 0,00E+00 | **24b_1_** | 0,00 | 0,00E+00 | 0,00E+00 | 0,00E+00 |
| **5b_2_** | 0,00 | 0,00E+00 | 0,00E+00 | 0,00E+00 | **24b_2_** | 0,00 | 0,00E+00 | 0,00E+00 | 0,00E+00 |
| **9a_1_** | 0,00 | 0,00E+00 | 0,00E+00 | 0,00E+00 | **25a_1_** | 0,00 | 0,00E+00 | 0,00E+00 | 0,00E+00 |
| **9a_2_** | 0,00 | 0,00E+00 | 0,00E+00 | 0,00E+00 | **25a_2_** | 38,36 | 5,18E+00 | 1,73E+00 | 7,33E+01 |
| **9b_1_** | 0,00 | 0,00E+00 | 0,00E+00 | 0,00E+00 | **25b_1_** | 0,00 | 0,00E+00 | 0,00E+00 | 0,00E+00 |
| **9b_2_** | 0,00 | 0,00E+00 | 0,00E+00 | 0,00E+00 | **25c_1_** | 36,96 | 1,38E+01 | 4,58E+00 | 1,94E+02 |
| **10a_1_** | 0,00 | 0,00E+00 | 0,00E+00 | 0,00E+00 | **26a_1_** | 0,00 | 0,00E+00 | 0,00E+00 | 0,00E+00 |
| **10a_2_** | 0,00 | 0,00E+00 | 0,00E+00 | 0,00E+00 | **26a_2_** | 0,00 | 0,00E+00 | 0,00E+00 | 0,00E+00 |
| **10b_1_** | 0,00 | 0,00E+00 | 0,00E+00 | 0,00E+00 | **26b_1_** | 0,00 | 0,00E+00 | 0,00E+00 | 0,00E+00 |
| **10b_2_** | 0,00 | 0,00E+00 | 0,00E+00 | 0,00E+00 | **26b_2_** | 38,20 | 5,80E+00 | 1,93E+00 | 8,20E+01 |
| **16_1_** | 0,00 | 0,00E+00 | 0,00E+00 | 0,00E+00 | **27a_1_** | 39,15 | 2,98E+00 | 9,93E-01 | 4,21E+01 |
| **16_2_** | 0,00 | 0,00E+00 | 0,00E+00 | 0,00E+00 | **27a_2_** | 0,00 | 0,00E+00 | 0,00E+00 | 0,00E+00 |
| **19** | 39,43 | 2,45E+00 | 8,18E-01 | 3,47E+01 | **27b_1_** | 37,38 | 1,03E+01 | 3,43E+00 | 1,45E+02 |
| **20a_1_** | 0,00 | 0,00E+00 | 0,00E+00 | 0,00E+00 | **27b_2_** | 0,00 | 0,00E+00 | 0,00E+00 | 0,00E+00 |
| **20a_2_** | 0,00 | 0,00E+00 | 0,00E+00 | 0,00E+00 | **27c_2_** | 38,00 | 6,64E+00 | 2,21E+00 | 9,38E+01 |
| **20b_1_** | 0,00 | 0,00E+00 | 0,00E+00 | 0,00E+00 | **29a_1_** | 36,89 | 1,45E+01 | 4,83E+00 | 2,05E+02 |
| **20b_2_** | 0,00 | 0,00E+00 | 0,00E+00 | 0,00E+00 | **29a_2_** | 0,00 | 0,00E+00 | 0,00E+00 | 0,00E+00 |
| **21a_1_** | 0,00 | 0,00E+00 | 0,00E+00 | 0,00E+00 | **29b_1_** | 37,91 | 7,09E+00 | 2,36E+00 | 1,00E+02 |
| **21a_2_** | 0,00 | 0,00E+00 | 0,00E+00 | 0,00E+00 | **29b_2_** | 0,00 | 0,00E+00 | 0,00E+00 | 0,00E+00 |

### *2.10 Tannerella forsythia*

#### Standard dilutions and standard curve of T. forsythia

| **Log(Copy's/reaction)** | **Copy's/reaction** | **Cq** |
| --- | --- | --- |
| 1,04E+01 | 2,69E+10 | 18,25 |
| 9,43E+00 | 2,69E+09 | 20,83 |
| 8,43E+00 | 2,69E+08 | 24,61 |
| 7,43E+00 | 2,69E+07 | 29,79 |
| 6,43E+00 | 2,69E+06 | 32 ,19 |
| 5,43E+00 | 2,69E+05 |  |

| 4,43E+00 | 2,69E+04 |  |
| --- | --- | --- |
| 3,43E+00 | 2,69E+03 |  |

####

####

####

####

####

####

####

####

####


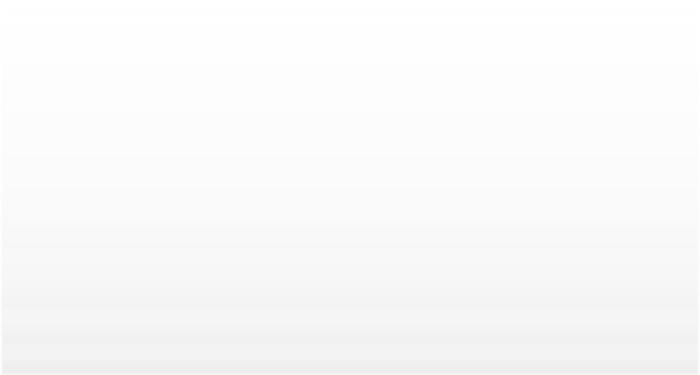

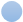

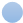

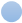

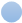

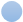


#### y =

#### -

#### 3

#### 56,203

#### ,6855x +

#### R² = 0,9859

#### 0

#### ,

#### 00

#### 00

#### 5

#### ,

#### ,

#### 10

#### 00

#### 15

#### 00

#### ,

#### ,

#### 20

#### 00

#### 25

#### ,

#### 00

#### 00

#### ,

#### 30

#### 35

#### ,

#### 00

#### 00

#### ,

#### 40

#### ,

#### 45

#### 00

#### 00

#### ,

#### 50

#### 1

#### ,

#### 00E+00

#### 3

#### ,

#### 00E+00

#### 5

#### ,

#### 00E+00

#### 7

#### ,

#### 00E+00

#### 9

#### ,

#### 00E+00

#### ,

#### 10E+01

#### 1

#### ^Cq^

#### Log (copy's/reaction)

#### Standard curve

#### Cq-value, detections in template, number of T. forsythia in template and number of T. forsythia /mL

| **Sample** | Cq | Detections  in  template | Number of MO in template | Number of MO/ml | **Sample** | Cq | Detections  in  template | Number of MO in template | Number  of  MO/ml |
| --- | --- | --- | --- | --- | --- | --- | --- | --- | --- |
| **AD** | 0,00 | 0,00E+00 | 0,00E+00 | 0,00E+00 | **21b_1_** | 0,00 | 0,00E+00 | 0,00E+00 | 0,00E+00 |
| **AD** | 0,00 | 0,00E+00 | 0,00E+00 | 0,00E+00 | **21b_2_** | 0,00 | 0,00E+00 | 0,00E+00 | 0,00E+00 |
| **AD** | 0,00 | 0,00E+00 | 0,00E+00 | 0,00E+00 | **21c_1_** | 0,00 | 0,00E+00 | 0,00E+00 | 0,00E+00 |
| **AD** | 0,00 | 0,00E+00 | 0,00E+00 | 0,00E+00 | **21c_2_** | 0,00 | 0,00E+00 | 0,00E+00 | 0,00E+00 |
| **1** | 0,00 | 0,00E+00 | 0,00E+00 | 0,00E+00 | **22a_1_** | 0,00 | 0,00E+00 | 0,00E+00 | 0,00E+00 |
| **2** | 32,18 | 1,65E+04 | 8,24E+03 | 3,29E+05 | **22a_2_** | 0,00 | 0,00E+00 | 0,00E+00 | 0,00E+00 |
| **3a_1_** | 38,11 | 4,06E+02 | 2,03E+02 | 8,12E+03 | **22b_1_** | 0,00 | 0,00E+00 | 0,00E+00 | 0,00E+00 |
| **3a_2_** | 37,72 | 5,16E+02 | 2,58E+02 | 1,03E+04 | **22b_2_** | 0,00 | 0,00E+00 | 0,00E+00 | 0,00E+00 |
| **3b_1_** | 0,00 | 0,00E+00 | 0,00E+00 | 0,00E+00 | **23a_1_** | 0,00 | 0,00E+00 | 0,00E+00 | 0,00E+00 |
| **3b_2_** | 0,00 | 0,00E+00 | 0,00E+00 | 0,00E+00 | **23a_2_** | 0,00 | 0,00E+00 | 0,00E+00 | 0,00E+00 |
| **4a_1_** | 37,11 | 7,56E+02 | 3,78E+02 | 1,51E+04 | **23b_1_** | 0,00 | 0,00E+00 | 0,00E+00 | 0,00E+00 |
| **4a_2_** | 38,66 | 2,87E+02 | 1,44E+02 | 5,74E+03 | **23b_2_** | 0,00 | 0,00E+00 | 0,00E+00 | 0,00E+00 |
| **4b_1_** | 0,00 | 0,00E+00 | 0,00E+00 | 0,00E+00 | **23c_2_** | 0,00 | 0,00E+00 | 0,00E+00 | 0,00E+00 |
| **4b_2_** | 0,00 | 0,00E+00 | 0,00E+00 | 0,00E+00 | **23d_2_** | 0,00 | 0,00E+00 | 0,00E+00 | 0,00E+00 |
| **5a_1_** | 19,77 | 3,85E+07 | 1,92E+07 | 7,70E+08 | **24a_1_** | 30,50 | 4,72E+04 | 2,36E+04 | 9,45E+05 |
| **5a_2_** | 35,10 | 2,65E+03 | 1,33E+03 | 5,31E+04 | **24a_2_** | 28,22 | 1,95E+05 | 9,77E+04 | 3,91E+06 |
| **5b_1_** | 0,00 | 0,00E+00 | 0,00E+00 | 0,00E+00 | **24b_1_** | 37,61 | 5,55E+02 | 2,77E+02 | 1,11E+04 |
| **5b_2_** | 37,68 | 5,32E+02 | 2,66E+02 | 1,06E+04 | **24b_2_** | 0,00 | 0,00E+00 | 0,00E+00 | 0,00E+00 |
| **9a_1_** | 0,00 | 0,00E+00 | 0,00E+00 | 0,00E+00 | **25a_1_** | 25,47 | 1,09E+06 | 5,45E+05 | 2,18E+07 |
| **9a_2_** | 0,00 | 0,00E+00 | 0,00E+00 | 0,00E+00 | **25a_2_** | 0,00 | 0,00E+00 | 0,00E+00 | 0,00E+00 |
| **9b_1_** | 0,00 | 0,00E+00 | 0,00E+00 | 0,00E+00 | **25b_1_** | 0,00 | 0,00E+00 | 0,00E+00 | 0,00E+00 |
| **9b_2_** | 0,00 | 0,00E+00 | 0,00E+00 | 0,00E+00 | **25c_1_** | 0,00 | 0,00E+00 | 0,00E+00 | 0,00E+00 |
| **10a_1_** | 0,00 | 0,00E+00 | 0,00E+00 | 0,00E+00 | **26a_1_** | 0,00 | 0,00E+00 | 0,00E+00 | 0,00E+00 |
| **10a_2_** | 0,00 | 0,00E+00 | 0,00E+00 | 0,00E+00 | **26a_2_** | 0,00 | 0,00E+00 | 0,00E+00 | 0,00E+00 |
| **10b_1_** | 0,00 | 0,00E+00 | 0,00E+00 | 0,00E+00 | **26b_1_** | 0,00 | 0,00E+00 | 0,00E+00 | 0,00E+00 |
| **10b_2_** | 0,00 | 0,00E+00 | 0,00E+00 | 0,00E+00 | **26b_2_** | 0,00 | 0,00E+00 | 0,00E+00 | 0,00E+00 |
| **16_1_** | 0,00 | 0,00E+00 | 0,00E+00 | 0,00E+00 | **27a_1_** | 0,00 | 0,00E+00 | 0,00E+00 | 0,00E+00 |
| **16_2_** | 0,00 | 0,00E+00 | 0,00E+00 | 0,00E+00 | **27a_2_** | 0,00 | 0,00E+00 | 0,00E+00 | 0,00E+00 |
| **19** | 0,00 | 0,00E+00 | 0,00E+00 | 0,00E+00 | **27b_1_** | 0,00 | 0,00E+00 | 0,00E+00 | 0,00E+00 |
| **20a_1_** | 44,41 | 7,93E+00 | 3,96E+00 | 1,59E+02 | **27b_2_** | 0,00 | 0,00E+00 | 0,00E+00 | 0,00E+00 |
| **20a_2_** | 0,00 | 0,00E+00 | 0,00E+00 | 0,00E+00 | **27c_2_** | 0,00 | 0,00E+00 | 0,00E+00 | 0,00E+00 |
| **20b_1_** | 0,00 | 0,00E+00 | 0,00E+00 | 0,00E+00 | **29a_1_** | 0,00 | 0,00E+00 | 0,00E+00 | 0,00E+00 |
| **20b_2_** | 0,00 | 0,00E+00 | 0,00E+00 | 0,00E+00 | **29a_2_** | 0,00 | 0,00E+00 | 0,00E+00 | 0,00E+00 |
| **21a_1_** | 0,00 | 0,00E+00 | 0,00E+00 | 0,00E+00 | **29b_1_** | 0,00 | 0,00E+00 | 0,00E+00 | 0,00E+00 |
| **21a_2_** | 0,00 | 0,00E+00 | 0,00E+00 | 0,00E+00 | **29b_2_** | 0,00 | 0,00E+00 | 0,00E+00 | 0,00E+00 |
